# Spirit of the Foreign Periodicals, &c.

**Published:** 1844-10-01

**Authors:** 


### Spirit of the Foreign Periodicals.

### Death of M. Geoffroy St. Hilaire.

After a long and distinguished public career, this eminent philosopher was gathered to his fathers on the 22nd of June last. His funeral was attended by an immense number of the leading *Savans* of the French Metropolis, and many touching and eloquent orations were made over his grave.

The cords of the pall were held by Baron *Dupin*, President of the Academy of Sciences; M. *Dumas*, Dean of the Faculty; M. *Ferrus*, President of the Academy of Medicine; and M. *Chevreul*, Director of the Museum of Natural history. Among the mourners were MM. *Arago*, *Flourens*, *Dumeril*, *Mathieu*, *Jomard*, *Rayer*, *Blainville*, *Pariset*, *David*, *Victor Hugo*, &c. When the procession had reached the gates of the Pere la Chaise Cemetery, a number of the work-people belonging to the Jardin des Plantes, spontaneously unyoked the horses, and drew the hearse to the place of sepulture—a touching homage to the worth of the deceased.

M. *Dumeril*, the organ of the Zoological section of the Academy, recapitulated, in language of noble simplicity, the great services which M. *St. Hilaire* had conferred on Comparative Anatomy and Zoology. M. *Chevreul* then spoke very feelingly in the name of the Museum, pointing out the deep obligations it was under to the zeal and generosity of the Founder of the Menagerie. M. *Dumas* and *Pariset* followed; the former recorded the high merits of the illustrious author of Philosophic Anatomy as one of the professors to the Faculty of Sciences; and the latter expressed, in the beautiful language which always characterises his style, the solemn regrets of the Academy of Medicine, which had the honour of ranking the deceased among its Associate Members. To these succeeded M. *Serres*, who, in a strain of heartfelt eloquence, proclaimed those lofty ideas of Unity of Composition, of Organic Analogues, and of the laws of Compensation in Animal Development, with which the name of *St. Hilaire* will ever be associated.

The following passages from several of these funeral orations will be found to touch upon the most memorable events in the scientific life of this venerable student of nature.

"Death, in striking down *Stephan Geoffroy Saint-Hilaire*, has at length removed from the scene the last of those administrative professors of the Museum, whose nomination dates so far back as the 10th of June, 1793—the epoch of the re-organisation of the Museum of Natural History. Three months previously, under the auspices of the venerable *Daubenton*, and in the 21st year of his age, he had been admitted as sub-keeper and demonstrator—not of Zoology, of which hitherto he knew scarcely anything—but of Mineralogy in this establishment. How came it, it may be here asked, that a man, who for half a century was entirely engaged in the study of organised and living matter, without recoiling before the most abstruse and profound speculations, should have commenced his career as a Mineralogist? The fact is that it was the result of the pressing recommendation of the fellow-labourer of *Buffon*, the famous *Abbé Hany*, who, having instructed the young *Hilaire* in the knowledge of Crystallography, had formed the highest esteem for his talents and character. The pupil, ere long, had a memorable opportunity of testifying his gratitude to his friend and teacher. The *Abbé*, falling under the suspicions of the Convention that was then in the plenitude of its power, was cast into prison, and would probably have fallen a sacrifice to the popular fury, but—thanks to the noble daring of the young *St. Hilaire*—he, with some other ecclesiastics, made their escape from their dismal confinement before the fatal 2nd and 3rd of September. This act of generous devotion soon received a worthy recompense, by opening up to the youthful student's ambition the career in which he afterwards so highly distinguished himself. By the patronage of *Hany*, *Daubenton*, and *Lacapede*, he was elected their colleague at the Museum, although he had not yet passed the 21st year of his age.

"At first he devoted his attention, with the most unwearied zeal, to the examination of Vertebrate Animals, and so assiduously did he pursue his studies that, in less than four years after his appointment, he had already grouped and arranged the numerous collections of the Museum. He had moreover published a Memoir, in which the idea of the *unity of Organic formation* is clearly announced as a result of his anatomical researches. It was he that set the example of keeping live animals at this national establishment, so that not only their habits might be watched, but also the connection between these habits and the organisation of their bodies, as revealed by dissection, might be more accurately determined.

"Such was the commencement of that Menagerie, which, under the sole direction of M. *St. Hilaire*, has become the model of an institution that is the envy of every nation in Europe.

"Joining his acquirements and efforts to those of his friend and fellow-labourer, *George Cuvier*—whom he had invited to Paris—he became with him one of the founders of the Natural Classification of the first two families or groupes in the Zoological series, the Mammalia and the Birds.

"Animated by an enthusiastic love of his favourite science, he was one of that noble band of *Savans*, who accompanied Buonaparte to Egypt, and shared in all the dangers and glories of that memorable campaign. He travelled as far as the Cataracts of the Nile, exploring with the utmost zeal all the natural productions of the country through which he passed. His name is inscribed in the pages of that brilliant episode of our history, associated with those of *Denon*, *Larrey*, &c.

"After the capitulation of Alexandria, when the English Commissioner claimed, in virtue of the terms of the treaty that had been formed between the two armies, the possession of all the scientific collections that had been made by the different Savans in the land of the Pharaohs, *Geoffroy St. Hilaire*, by a bold and eloquent appeal, succeeded in preserving his collection to his own country; and thus, by his energy and courage, he was the means of enriching the galleries of France with some of the most interesting *souvenirs* of the Egyptian Expedition."

"In 1808, he was appointed by Napoleon to execute the duties of a delicate mission to Portugal; and he succeeded admirably in effecting the object of his journey. Another person might have availed himself of the privileges of war to obtain possession of the rich collections of Natural History at Ajuda; but *St. Hilaire*, in accordance with the laws of the most strict justice, did not so abuse the power entrusted to him. All that he required was a mutual exchange, that could not fail to be of advantage to both countries; and thus the duplicate specimens of the Portuguese collection were obtained to enrich that of the French Museum, and those of our establishment served to fill up in part the deficiencies of the Ajuda Gallery.

"The events of 1815 brought into conspicuous view the tact, as well as the justice, that had regulated our compatriot's conduct; for at this period, when France was a second time over-run by the armies of the Coalition, the Portuguese minister declared to the Duke of Richelieu that he had no demands to make against the French government for the specimens which had been withdrawn, seven years before, from the museums of his country; expressly declaring that they had not been taken away by any force of conquest, but had been readily exchanged for others that were now the property of Portugal."

(It is pleasing to find a French writer in the present day protesting, although in an indirect manner, against the robbery of the works of art and science that was perpetrated on a grand scale by his fellow-countrymen, during the wars of the Republic and Empire.)

"The science of organic relations and analogies in Anatomy, of which a few sparks did shine forth in some parts of *Buffon's* writings, was checked, and, as it were, drowned in the wearisome details with which *Daubenton* had subsequently overlaid it. It was *Geoffroy* who, by one of those bold and penetrating glances of genius which give rise to a new era in natural knowledge, disengaged it from the trammels of a long-established custom.

"It was when our illustrious countryman was seeking to explain to himself the mechanism of the bony head in the fish, and to reduce the numerous pieces, of which it consists, to the known type of the cranium in vertebrated animals, that he first caught a glimpse of that curious and most interesting Law of Organisation which he afterwards worked out in so beautiful a manner.

"When the happy thought suggested itself to him of comparing the various pieces, of which the cranium in the fish is composed, with the osseous nuclei in the head of the human embryo, it may be truly said that he took the first, the initiatory step in that memorable career, which subsequently led to the elucidation of a multitude of questions that had hitherto puzzled and perplexed all previous Naturalists and Comparative Anatomists."

"It is to *Geoffroy St. Hilaire* that we owe the important discoveries that the Embryo of animals is not primarily the mere miniature copy of their perfect and mature development; and that, before an animal assumes the permanent form of its organisation, it passes through a series of forms which are of intermediate and of transitory existence.

"It is to him that Comparative Embryology—so long neglected because it seemed to be almost objectless—has of recent years become one of the very fundamental parts of Zoogeny—that indeed which commands and over-rules all the rest.

"The earth becomes to our contemplation a vast laboratory, where there is continually forming a succession of new beings in a progressive and ascending scale, and all of which are linked together in an unbroken chain, from the Infusoria, the starting-point of nature, up to the Mammalia and to Man himself, the highest limit of its efforts:—a thought, the depth and wisdom of which are every day confirmed by the discoveries of Palæontology. According to this view the entire animal kingdom is regarded as a single being; which, during its formation, is arrested at various periods of its development, and thus determines, at each epoch of its interruption, the distinctive characters of classes, families, genera and species."

M. *St. Hilaire* appears to have been truly happy in the social, and especially in the domestic, relations of life. For many years before his death he had become entirely blind; but he always retained a serenity and cheerfulness of character that endeared him to all his friends. The affection of a devoted wife, and the solicitude of a fond daughter were ever near, ready to soothe and comfort him:—

"After having received," says one of his panegyrists, "so much instruction from this soul in its strength, it only remained that we should learn of him how we ought to die. Like *Galileo*, he had lost his sight; but the calmness of his disposition was never ruffled for a moment; he still smiled at the mighty wonders of Creative Wisdom in Heaven and in Earth, which he still saw and dwelt upon with the eyes of his mind. Such was the peace of a man, who had long accustomed himself to meditate on the secret plans of an over-ruling Providence. What is more sublime than this death of genius, which, when so directed and controlled, is the very sanctity of the intellectual powers? With smiles he approached to the very fountain of truth, and joyfully awaited the hour of his departure. A few days before his death, when thankful for the kind attention of domestic love that watched over his bed, he exclaimed, 'Je suis presque heureux d'etre aveugle.' Happy old man! Who is there among us who does not long for such an end."

"Twenty years ago, when a stranger entered our Academy of Sciences, he could not fail to be struck with a sort of holy respect, on seeing around him so many men of high genius, the glory of France, and the luminaries of their age. There the geometrician might behold *Laplace, Ampere, Legendre,* and *Poisson;* there the student of physical science saw *Hany, Berthollet, Chaptal,* and *Vauquelin,* and the naturalist met with *Jussieu* and *Desfontaines, Cuvier* and *Geoffroy St. Hilaire.*

"All these men, who had sprung up during the time of the Republic, and whom the Empire had promoted to dignity and distinction; men of iron for labour, and of fire for thought, and whose discoveries have supported, but without paling, the brilliancy of the contemporaneous achievements of policy and war—all have at length disappeared from among us; and this grave, at which we now stand, is about to close over one of the most distinguished of their number. In this day of mourning alike of science and of our country, may our souls be lifted up to the contemplation of those great men who, in the course of a few years, have vanished from our sight; and may the rising generation learn to understand that, in the absence of those social crises which, though terrible for the time, yet work a salutary effect on the human race, they may still find, in the fulfilment of the calm duties of life, and in the prosecution of scientific and literary pursuits, inspirations as elevating and hopes as dignified as ever kindled the breasts of those who have gone before them!"

### RE-INTERMENT OF BROUSSAIS.

The remains of *Broussais* were recently exhumed from the Cemetery at Pere la Chaise to be deposited under the statue, which has been erected to his memory at the Val-de-Grace. As a matter of course, there was another imposing ceremony got up on the occasion, and deputations from a variety of learned Societies attended, to renew their respectful homage to the Napoleon of Medicine. Two funeral orations were pronounced, before the body was lowered "dans ses pénates eternels." Here is the exordium of the first. "Although the ceremony, which has brought us together this day, is not exempt from all the impression of funeral recollections, yet there now prevails in our bosoms a sentiment of pious joy, like that which attends the consecration of a glorious name, or the return of the head of a family to his home, after the absence of many years. The remains of M. *Broussais* are at length committed to their proper resting-place; for the true abode of the distinguished dead is in that place which they had adopted by their love during life, and which they have rendered famous by their own imperishable renown." . . . . . . . "For the last time, adieu, *Broussais!* In separating ourselves from thee, receive anew the expression of those sincere regrets which thy loss has inspired, and which are only soothed by the thought that one of our professors still worthily bears the glorious name which thou hast delegated to him."

### Berzelius on the Urine.

The distinguished Swede, after giving a most elaborate account of the composition of this animal fluid, proceeds to point out how much its composition is affected by a variety of substances when taken into the stomach. The following examples will abundantly prove the truth of this remark.

After the free use of mercurial ointment, the Urine is found to contain salts of this metal in minute quantities. To detect their presence, we have only to dry the sediment that is precipitated, and then to calcine it: globules of mercury may thus be obtained.

Nitre, the yellow Prussiate of Potash, and many metallic salts, especially those of Iron, may readily be discovered in the urine, not long after they have been swallowed.

After the employment of large quantities of any ferruginous preparation, the urine sometimes acquires a feeble blueish or greenish hue—owing, says *Berzelius*, to the union of the Iron with the ferro-cyanic Acid, which may be generated by the decomposition of different animal matters within the body itself.

Soon after Tartaric or Oxalic Acid has been taken into the stomach, the urine often deposits, as it cools, oxalate or tartrate of Lime—a deposit that is increased by the addition of the chloruret of lime to the fluid. The Malic, Citric, and Tartaric Acids render the urine more or less decidedly acid. Succinic Acid also re-appears in the urine. Not so, however, with the Benzoic Acid; for this seems to become transformed within the organism into Hippuric Acid—according to the observations of *Wohler, Boyé,* and *Leaming.*

The infusion of Nut-Galls is known to pass into the urine; for a black precipitate is found to be formed on the addition of a ferruginous solution. After the administration of Iodine, the iodurets of Potash and Ammonia are discoverable. The alkaline Carbonates also, the Borates, the Silicates, and the Chlorates may be detected by the addition of their respective re-agents. The same holds true of the yellow Prussiate of Potash: the red prussiate is converted into the yellow. The Sulphuret of Potassium is absorbed in part only, without alteration; part becoming oxydised during the circulation, and converted into a sulphate.

The Vegetable Salts, having Potash and Soda for their bases, are transformed into Carbonates; for the urine is then found to be alkaline, and to effervesce on the addition of an acid. The same phenomenon is often observed when a person has been eating very freely of certain fruits, as apples, cherries, strawberries, and raspberries, which contain the malate or the citrate of potash. This fact explains the utility of these fruits occasionally as a remedy in uric acid gravel.—*Journal de Pharmacie.*

---

### Mineral Salts, taken by the Mouth, discoverable in the Urine.

Dr. *Kramer*, professor of chemistry in the university of Milan, read, in the course of last year, before the Imperial Institute of Lombardy, an elaborate account of his researches for the purpose of discovering in the Blood, the Urine, and other products of animal Secretions, various mineral Salts that had been taken into the stomach.

It would be tedious to report the details of his experiments; all that we propose to do at present is, to present the most important conclusions which he has deduced from his extensive and very carefully conducted investigations.

1. The salts with alkaline bases, which we are in the habit of administering to man and the lower animals, pass with facility into the blood, urine, and even—this holds true more especially with the Ioduret of Potassium—into the sweat and saliva.

2. The blood and urine, when once charged with alkaline salts, free themselves of these foreign matters with very rapid progression.

3. The Salts of Barytes (at least the Chloruret) pass in small quantities into the blood and urine: it is, however, difficult to detect their presence by the ordinary chemical re-agents.

4. The vapour of certain substances, when inhaled into the throat, are absorbed and pass into the circulating fluids with a truly remarkable rapidity; as is proved by experiments with the Vapour of Iodine, traces of which are discoverable in the blood in half an hour after the commencement of the inhalation.

5. Very many of the Compounds of the Metals, properly so called, with other substances, pass into the blood and urine; the presence of the metal may generally be discovered in both fluids. The metallic salts, which have been chiefly subjected to experiment, are the sulphate of Mercury, the sulphuret of Antimony, the tartrate of Antimony, the muriate of Silver, the Carbonate and Sulphate of Iron, and, lastly, the various Combinations of Copper.

6. Some of the metallic Salts and Compounds—for example, those of Copper—may even be detected in the blood and urine eight or ten days after the internal use of the preparation has been quite suspended.

7. Iron, administered by the mouth, is rapidly absorbed and passes into the blood and urine: it should be borne in mind, however, that the urinary secretion in health contains a minute quantity of this metal.

8. Copper also is found, in a still more minute quantity in healthy urine; but it seems probable that the existence of this metal in animal fluids is derived from the copper vessels employed in domestic economy, and also from not a few articles that are used for food. If copper is present in the urine, we may fairly suppose that it may also exist in the blood.

9. Normal Blood always contains a certain quantity of Manganese: the urine also does not appear to be quite exempt from it.—*Giornale dell'Instituto Lombardo.*

*Remarks.*—The rapid absorption of so many metallic and other salts into the animal economy is a subject of great interest to the practical physician, as well as to the mere physiologist. It suggests, among other considerations, the necessity of more caution, on the part of medical practitioners, than is generally used in the administration of these substances. How must the system become thoroughly impregnated with such a metal as Mercury, when the patient has been taking blue-pill, for example, two or three months without intermission! One might almost believe the assertion of the once famous St. John Long, that he could extract globules of pure mercury from the skin of some of his patients.—*Rev.*

---

### M. MALGAIGNE ON THE ABUSE OF TENOTOMY IN VARIOUS DEFORMITIES.

Our readers are probably aware that the subject of M. *Guerin's* alleged almost invariable success in the treatment of even the most unpromising deformities, by the operation of dividing those tendons and muscles which are supposed to be contracted, has been recently brought before the Royal Academy—of which he is a member—by M. *Malgaigne,* and that the discussion thereon ensuing has been carried on with no little asperity by both parties. We have already more than once expressed our own opinion as to the merits of the "grand orthopœdiste:" we shall now select a few passages from a recent paper by M. *Malgaigne*, in which he comments upon and criticises the doctrines and practice of his opponent. * * * * * *

Suppose, says M. *Guerin*, that a surgeon has to treat a case of deformity of the foot, when all the muscles, with the exception of two or three, are in a state of paralysis. Those, which are not paralysed, are, as a matter of course, retracted, and draw the foot in the direction of their action. If we cannot indeed effect a cure of the paralysis by Tenotomy, we can, and we ought to, correct the vicious direction of the foot by dividing the tendons which occasion it. *Cela est fait.* Forthwith the critic launches his thunders against the operator and the operation, on the ground that he never heard of Palsy being treated by the division of the tendons of the affected part! We may here observe that, in the same limb, there are not unfrequently some muscles which are palsied, while others are retracted; and therefore surely we may fairly presume that the one set may be divided, while at the same time we endeavour to excite the other set to action.

"Now we beg to offer a few remarks on the doctrine here inculcated. The first point to be determined is to ascertain what the author really means by *retracted* muscles. Does he allude to the normal and physiological retraction, which always exists in healthy muscles when opposed to such as are paralysed? We may fairly infer, from the above-quoted paragraph, that such is his meaning; and it is very certain that, in the instance of the paralytic foot to which we have alluded, the tendo Achillis was divided by M. *Guerin* for the simple retraction of the muscles of the extremity. If such be the case, the doctrine here propounded is so utterly inconsistent with all the sound principles of enlightened surgery, that we need not surely waste a line in combating its error. Never will any muscle, when acting by its physiological action alone, prevent the foot from being reduced to its normal direction by the effort of the hand, or by the application of a boot. If you divide the tendon under such circumstances, what good do you expect to result from it? Either a re-union of the divided ends will take place, and the muscle will recover its contracting power; or this power will be entirely lost, and then, in place of a partial, you will have a complete, paralysis. Is this a result to be desired?

"Let us see what was the result of such a line of practice in a case reported by M. *Guerin.*

"A child, six years old, was some time ago admitted into the Infant Hospital with the following deformity:

"In the second year of her age, she had been seized with convulsions, and these had been followed by palsy of the left leg. M. *Bouvier*, who was consulted about this time, recommended the use of a spring-boot with the view of supporting the weak extremity, advising at the same time that the case should be left to the natural restorative efforts of the constitution. About twelve months afterwards, the paralysis had very sensibly diminished; but the foot still hung down and was drawn somewhat outwards. M. *Guerin*, who now saw her, pronounced the case to be one of *pied-bot valgus*, and, (according to the statement of the child's mother) he at once proceeded to divide the tendo Achillis. On the young creature being discharged from the hospital some time afterwards, he assured the mother that her child would be certainly cured of the club-foot, but perhaps not of the paralysis. Now, so far from this being the case, what is the state of the child at the present moment? The foot is precisely in the same condition as it was before the operation; the gait is excessively unsteady; the spine has begun to be curved; the affected leg is somewhat shortened, and the size of its calf is considerably less than that of the other leg. It must be quite unnecessary to comment on the practice pursued in this case: it was irrational, and therefore altogether unjustifiable. After no little *consideration of the subject*, I do not hesitate to assert, as a principle of surgical practice to which there is no exception, that it is utterly irrational to divide healthy muscles in demi-paralysed limbs."

M. *Malgaigne*, notwithstanding this assertion, willingly admits that, in certain cases of pathological contraction of a limb, the section of the retracted tendons is quite justifiable, and may be productive of much good. For example, if the Gastrocnemii muscles are in a state of such rigid contraction that the heel cannot be brought down to the ground, or the foot cannot be easily incased in an ordinary boot, the section of the tendo Achillis may be perfectly warrantable, as the patient may thereby be enabled to walk with much greater ease and comfort than he could before. The same may be affirmed of a case where the knee-joint has become flexed, in consequence of the predominance of the action of the flexor over that of the extensor muscles of the leg; for surely it is better, if a patient must have an anchylosed knee-joint, that the limb should then be in an extended than in a bent position.

"In January 1840, M. *Guerin* informed the Academy of Sciences that he had, three weeks before, divided the biceps, semi-tendinosus, semi-membranosus and rectus internus muscles on each side, in a young girl, for an incomplete luxation of the knee-joint. On the day after the operation, the deformity, we were told, was reduced to a simple normal flexion of the part; and, after the patient had been kept in the Orthopœdic Hospital for nine months, the report proceeded to state that the cure was complete, with the exception only of a certain degree of permanent flexion of the articulation.

"Now for the *real* history of this case. The girl, when seven years of age, had been seized with convulsions; after one of the violent attacks of which, the contractions and paralysis, with which she is now affected, were first observed. A variety of means were tried for her relief; but they all failed; and she was therefore sent to the Salpetrière as a poor helpless creature. M. *Mitivier*, touched with compassion at her pitiable condition, recommended the case to the notice of M. *Guerin*, who admitted her into the Hospital of Children, and performed two tenotomic operations, in the first of which he divided thirteen, and in the second five muscles or tendons, for the relief of her various deformities. Now let us hear what was the actual condition of this poor creature, when she was sent back to the Salpetrière, after having been all but cured. (!) She was then, and still continues, as miserably helpless as ever; and, in addition to her helplessness, she now suffers a good deal of pain in the site of the wounds that were made in different parts of the body.

"Another memorable case, in which the great Orthopœdist divided no fewer than 42 muscles and tendons at one séance and 'sans desemparer,' was quite as little creditable to the veracity, as well as the judgement, of the operator. M. *Phillips*, who allowed himself at first to be so far misled by the alleged success as even to adopt a similar line of practice in an analogous case, has candidly confessed (in his Memoir, de la Tenotomie Sous-cutanée, p. 18) that, in neither instance, was the operation productive of any decided benefit."—*Journ. de Chirurg.*

*Remarks.*—So much for the performances of M. *Guerin*. As is the case with all great *claqueurs*, the actual performances of this gentleman fall far short of his professions. But the blame should not fall upon him exclusively; there is more than one scientific (!) quack in the list of the Royal Academicians. One of his most zealous assailants is a gentleman who actually recommended—aye too, and he put his recommendation in practice—the forcible extension, by means of a powerful mechanical contrivance, of semi-anchylosed joints. After the death of one or two patients, who were subjected to this horrible contrivance, the scheme was dropped.—*Rev.*

### Section of the Flexor Tendons of the Knee in White Swelling.

One of the most successful applications of Tenotomy is unquestionably that of dividing the flexor tendons in certain cases of diseased joints, with the view of insuring the perfect quietude of the articular surfaces. It has been tried by several surgeons in White-Swelling of the Knee, and, we rejoice to say, with very promising success.

M. *Ribes*, a man of great experience, has recorded his opinion of the practice in the following words:

"The medical art is rich in therapeutic remedies for the relief of White-swelling of the Knee-joint; but, in almost all cases, a simple cause has made them to prove utterly inefficient. This cause is the forced and permanent contraction of the flexor muscles of the leg. *Eh bien!* Why should we not perform, at the proper time, the subcutaneous section of the tendons of the Semi-membranosus, Semi-tendinosus and Biceps muscles, which keep up this uneasy state of things? By this easy operation, we may rationally hope not only to relieve the existing pain and distress, but also very materially to promote the formation of anchylosis, and consequently the cure of the disease. This simple and safe operation is already admitted and recognised by surgeons."

M. *Ribes* is one of those men whose name alone is worth a multitude of authorities, and whose simple approval will often weigh more than the most elaborate commendation of others.

---

### M. Rognetta on the Dynamic Treatment of Cancer.

Let us first enquire what is meant by the phrase of Cancerous Diathesis, and what is the nature of the dynamic condition that accompanies it. We have the choice of two doctrines or theories on the subject.

According to one of these, the existence of the diathesis in question precedes, in an occult manner, the formation of the local cancerous tumor; whereas, according to the other, it is the consequence and result of the absorption into the general system of the poisonous matter from that morbid growth. The late Baron *Boyer* maintained the first of these opinions, and insisted upon its adoption with great earnestness; but most of our leading metropolitan surgeons in the present day—*Roux, Velpeau, Gerdy, Lisfranc* and *Blandin*—reject the doctrine of primitive diathesis, and therefore regard the disease of Cancer as strictly local in its nature at first. The partisans on both sides appeal to the results of operations; but, alas! the conclusions, to which they arrive, are very different. The one party maintains that there is very generally a relapse after the diseased growth has been extirpated; while the other contends that such is not the case, if the operation be performed sufficiently early.

M. *Rognetta* seems to adopt an intermediate opinion; for we find him saying that, "it is perhaps more reasonable to admit that the local disease and the constitutional diathesis are developed simultaneously and proceed *pari passu*, and that the latter continues and advances after the tumor has been removed, just as much as when it is allowed to remain. There are even some reasons for believing that the re-action, which inevitably follows upon any severe surgical operation, must tend to accelerate the progress of the constitutional affection, if this be not counteracted by the administration of appropriate dynamic remedies. This perhaps is the reason why some practitioners of large experience have ultimately come to the conclusion of dissuading in all cases the removal of a truly cancerous tumor, in place of trying to find out any therapeutic means to arrest the advance of the morbid diathesis."

M. *Rognetta's* pathological views are partially expounded in the following passage: they appear to us to be in some points very fanciful.

"We do not say, with the Physiological School, that Cancerous disease is the product of Inflammatory action; but we are quite prepared to assert that the dynamic condition of this affection is in all its stages of a decidedly *hypersthenic* character, and also that it exhibits many points of resemblance with Chlorosis and Scrofula, more especially with the first of these morbid conditions. This assertion may seem strange; but we shall now endeavour to establish its probability.

"During the development of a cancerous growth, the system very generally will be found to exhibit symptoms of hypersthenic excitement; and, although the health may seem good, there is almost always a predisposition to inflammatory and congestive diseases. Be it remembered that a hypersthenic state of the body may exist without any actual excess in the quantity of the circulating fluid; and moreover, that this state, when it affects a great system,—the vascular tree, for example, as in Cancerous and Chlorotic patients—cannot be effectually got rid of under the mere influence of bleedings, whose action is but transient and of short duration. We must therefore have recourse to certain dynamic remedies, which exert an elective operation on the system that is chiefly implicated; and, when we have found such, we must persevere in their use for a very considerable length of time. We know that some eminent pathologists see nothing in Cancer and Chlorosis except mere affections of the blood: but this is certainly a great error. They mistake the consecutive effect for the primary disease, and, by following a false path of enquiry, they inevitably land in recommending a faulty practice."

As to the therapeutic part of the question, M. *Rognetta* seems to dwell chiefly on the high importance of diminishing and, if possible, subduing the hypersthenic condition of the vascular system, whether an operation for the removal of the cancerous tumor may be deemed expedient or not. It is from having neglected to attend to this constitutional affection before, as well as after, the performance of any surgical operation, that the disease is so often observed to return, even when every portion of the morbid texture has seemingly been utterly removed.

Our author carries his peculiar views so far that he even sees in the pallid hue of the skin, when the disease has reached its second stage, that of softening and ulceration, evidences of the hypersthenic excitement of the capillaries of the skin—the circulation in these vessels becoming gradually more and more completely extinguished, and consequently the process of assimilation, which depends upon their healthy condition, being quite interrupted.

"Examine," says he, "the large vessels, and you will find them hard, thick and resistent, as in all genuine hypersthenic affections; and moreover do we not always observe that everything, which excites the vascular system—as, for example, the use of a stimulating diet and spirituous liquors—very strikingly aggravates the state of such patients, and always increases their pallor and weakness?

"The icteric discolouration of the surface, in the more advanced stages of the disease, is the effect of the extreme decay of the organic functions and the consecutive impoverishment of the blood, and finally of the resorption of the cancerous matter into the system."

M. *Rognetta* gives sound advice when he insists upon a certain course of constitutional treatment to be adopted both for some time before, and for a length of time after, an operation has been performed. Whether he is right in his opinion that such treatment should invariably be directed to the correction of hypersthenic excitement—or, as our author expresses it, 'l'espece d'incendie devorant'—may, we think, be fairly disputed; but an objection on this score does not necessarily invalidate the general expediency of his therapeutic suggestions.

Among the *Hyposthenic* remedies that are most serviceable alike in Cancerous and in Chlorotic affections, he enumerates repeated small bleedings, also steel, quinine, hemlock, the aerated alkaline waters, acidulated drinks, warm baths, a mild unirritating diet, and the avoidance of all physical and moral excitement.

These remedies have never cured Cancer, it will be at once replied. True, but then it may be fairly asked in reply, have they ever been used with sufficient perseverance and discretion? Medical men have always been looking out for *specifics*; and they have rarely followed out any plan of constitutional treatment upon rational principles, for a sufficient length of time. When the disease has made considerable advances, the remedies which we have mentioned, although useful, cannot be expected to effect any very decided benefit; unless, indeed, they be so judiciously combined together, as to maintain the vascular system in an unvarying state of *hyposthenia*. Then we must also have recourse to the exhibition of more energetic means; as, for example, the preparations of Iodine, Arsenic, Mercury, &c.—which may be often very advantageously combined with the remedies of which we have already spoken.

We may here state, as a general rule, that these active poisonous drugs will always act more surely and decidedly, if administered in minute doses, and dissolved in a large quantity of fluid, so as to form a sort of mineral water. Thus given, they are much more readily absorbed into the system, and moreover, their irritative local action on the stomach and bowels is at the same time avoided.

The following practical remarks by M. *Tanchon*, who has paid so much attention to the pathology of Cancer, are well deserving of general notice.

"Mercury, Iodine, and Arsenic are the three remedies which I have most extensively used. Of these, Mercury is certainly the most potent sedative (*le plus hyposthenisant)* of the white or lymphatic vascular system that I know. By its aid, I have often taken the colour from the most sanguineous habits in the course of a few days. One of the best forms for using it is the Unguentum Neopolitanum or Pommade di Vigo, applied externally. Sometimes, however, the tolerance even of this simple form, on the part of the system, is by no means great; for I have seen it several times bring back engorgements which had already disappeared, and even produce pain, which it so generally soothes in a very remarkable manner.

"Iodine is an excellent remedy, when the stomach will bear its administration. The tincture is not a good form for giving it: an aqueous solution is greatly to be preferred. I occasionally use it as an outward application, combining small doses of Iodine in powder with starch, and then mixing this with Camphor or Belladonna, according to the indications of the case: this is what I call my *pulvero-topique*. The *ioduret* of Arsenic is the form in which I prefer to administer the latter active remedy. It should be given in very minute doses, and these very largely diluted. When the system bears, as it sometimes does, enormous doses of Arsenic without suffering, we may fairly presume either that the cancerous disease is incurable, or that the action of the remedy is directed on some other point of the economy."

So much for M. *Tanchon's* experience. According to M. *Rognetta's* observation, the Ioduret of Potassium is essentially a sedative of the vascular system. He condemns the use of Opium in all its forms; it acts, says he, almost always as a stimulant, and therefore cannot fail to prove injurious in Cancerous diseases.

Conium and Belladonna are not liable to the same objection. The former of these narcotics was certainly at one time vaunted too highly; nevertheless it is an excellent remedy, and should be used at a much earlier period of the disease than is usually done. M. *Rognetta* objects to the form of pill in which it is so generally exhibited; it would be decidedly more efficacious, if it were given in a fluid state, by being dissolved in a large quantity of water. In many cases it may be very serviceably associated with quinine, steel, and sometimes also with sulphur.

With respect to the propriety of removing a Cancerous tumor by a surgical operation, our author decidedly recommends that the sooner that this is done, the better;—"provided always that the constitution of the patient be treated dynamically both before and after the operation." For the purpose of effecting the removal he prefers the use of Caustics to that of the knife. His reasons we give in the following words:—

"If we consider that a chemical caustic acts slowly for several days in succession, becomes infiltrated pretty deeply into the flesh, passes in part into the healthy tissues, pursues the morbid product even into its most extreme recesses, is more or less completely absorbed, and acts as a general sedative, at the same time that it removes the mischief, bit by bit, (*à plusieurs reprises*) until a process of healthy granulation is established; if, moreover, we reflect that this destruction is accompanied with a dynamic *ataxia*, which the mineral poison produces upon the morbid part, and that the existence of this is an obstacle to the *hypersthenic* re-action of the general system—a re-action which is invariably the result of a cutting operation, and which, without fail, must accelerate the progress of the diathesis—we may readily understand the motives which have led me to prefer the use of caustics to that of the knife, whenever it is possible; or to join both means together, if such be ever deemed necessary. Experience has proved that more advantageous results have been obtained from the use of caustics than from that of cutting instruments; and we verily believe that these results would be still more favourable, if a judicious internal treatment had been adopted at the same time."—*Annales de la Therapeutique.*

---

### ON THE FORMATION OF THE AMMONIACO-MAGNESIAN PHOSPHATE IN PUTRESCENT MATTERS.

Numerous experiments have proved that, whenever any animal matter begins to putrefy, there are formed in it crystals of the ammoniaco-magnesian phosphate, which are discoverable by the use of the microscope. By this simple method they may be readily seen in putrescent urine, for example; but, not so, if this fluid be deprived of its fibrine by the addition of corrosive sublimate; then no trace of the crystals is to be found, even after four weeks of putrefaction.

If heat be employed to get rid of the coagulable contents, the crystals are formed only very slowly. They are abundant in the products of Nephritis, and Catarrh of the bladder. Dr. *Zimmermann* has discovered them also in the debris of carious teeth, in the exudation of pleuro-pneumonia, in the nasal mucus, in ordinary purulent matter, in the fæcal discharges—more especially of those animals which have a long intestinal canal, and which consequently retain for a length of time the food on which they live.

As often therefore as crystals of the Ammoniaco-magnesian Phosphate are found in any of the secretions of a patient, we may fairly presume that there exists a putrid decomposition of animal matter. It is only in this manner that we account for the presence of this salt in the alvine discharges in Typhus fever, and in the urine of persons affected with paralysis of the bladder.—*Wochenscrift fur die gesammte Heilkunde.*

### Cæsarian Operation on a Dead Woman: Child saved.

M. *Loweg* was called to the assistance of a pregnant woman, who had been long ill: she died very shortly after his reaching the house. The Cæsarian operation was immediately performed, and the child with the placenta was extracted without delay. It seemed on removal to be dead; but it had evidently continued to live up to the very moment of the mother's death. It was straightway put into a warm-bath, and artificial respiration was steadily employed. After a quarter of an hour's perseverance with these means, the pulsations of the heart were first discoverable, and soon afterwards the child began to breathe. It lived for several months.

*Remarks.*—It is indeed very rare that an infant, extracted from the uterus after the death of the mother, has been known to live. It deserves to be generally known that, when such has fortunately been the case, the success has usually been owing to the steady use for some time of the means employed in the present case; viz. of the warm bath and insufflation of the lungs by applying the lips directly to those of the infant, and breathing warm air into its chest —thereby keeping up an artificial respiration for a considerable period.—*Ibid.*

---

### Bicephalous Child: Ligature of One of the Heads.

On the 31st October, 1843, M. *Buhring* was called to see a new-born infant, on the back of whose head there was a large tumor, that was in a great measure covered with hair. On an attentive examination, he found that the child had a small head, having the forehead flattened as in the ape, the anterior fontanelle normal, the posterior one very large and covered with a thin skin; a part of the left parietal and of the corresponding occipital bone seemed to be awanting at the place where they join to form the lambdoid suture; the face was regularly formed, and the other parts of the body were well developed. On the occiput at the side, and a little to the right of the fontanelle, was situated the tumor, which was attached by a pedicle of an inch and a half in thickness; it seemed to be, as it were, a second head, that was actually larger than the true head of the child. This pseudo-head had the face turned to the right side; it had an ocular cleft, but without any eyeball, a projection in the site of the nose, and a depression in the place of the mouth. No distinct bony plate could be felt in any part; and indeed the round mass appeared to be formed by an elastic hairy scalp, which was of an almost cartilaginous firmness in some points. At the back part of this sort of Mole, there was a spheroidal mass, of a very red colour, evidently fluctuating, and yet insensible on pressure; although every other part of the tumor was so tender, that the infant began to cry on the gentlest touch. It was quite otherwise when the pedicle, which joined the two heads together, was compressed; there the sensibility was null; but symptoms of cerebral congestion appeared, if the constriction was increased beyond a certain degree.

M. *Buhring* determined to remove the tumor by ligature; but, first of all, he made an incision into the part which presented a sense of fluctuation. Between four and five ounces of a transparent yellow serosity were discharged; and, on separating the lips of the wound, he saw distinctly at its bottom two normal cerebral hemispheres, provided with regular medullary convolutions. When the ligature around the pedicle was tightened, the breathing of the infant, which had hitherto been quite tranquil, became hurried and laborious, the vessels of the face and head, especially the external jugular vein, very highly congested, and the pupils dilated. Some blood was therefore taken from the jugular vein, before the constriction was completed. When three ounces had flowed, all the accidents ceased, except some degree of embarrassment in the breathing, and certain convulsive twitches or irregular movements of the limbs. The child was applied to the breast, and it seemed to suck without inconvenience. After a few minutes had elapsed, the pseudo-head had become quite cold; and, as it was now completely destitute of feeling, M. *Buhring* had an opportunity of examining it at leisure. On making a longitudinal incision through the firm hairy scalp, he found a distinct *dura mater* underneath; and within this membrane a red-coloured medullary hemisphere, in which the great lobes and the crura of the brain were distinctly visible. During all the time of this anatomical examination, the infant continued to suck very quietly; and, for two hours afterwards, it did not exhibit the slightest appearance of suffering. The tumor was covered with pledgets dipped in a spirituous lotion, and these were kept on by means of a light bandage. The child lived for 36 hours after the operation.

*Dissection* left no doubt as to the nature of the monstrosity: it proved to be, beyond all doubt and question, a second head. The true head was completely organized: the connexion that existed between the two was not by any genuine medullary substance, but by nervous cords, blood-vessels, and a prolongation of *dura mater*. All the other organs were in a normal condition.—*Ibid, and Gazette Medicale.*

---

### ON THE RE-PRODUCTION OF THE CRYSTALLINE LENS.

The following is a summary of the conclusions to which M. *Textor*, in his recent dissertation, Ueber die Wiederzeugung der Krystallinse, has arrived.

The extraction or depression of the crystalline Lens is followed by the re-production of a new one that is more or less regular in its configuration, or at least by the formation of a certain portion of the crystalline mass.—This re-production is the work of the investing capsule, provided this remains uninjured.—When the capsule is removed, the lens is not re-produced.—The capsule adheres to the new crystalline ring (*bourrelet*), but not so very closely that they cannot be separated.—The newly-formed Lens possesses the same transparency as the old one.—The re-production of the Lens requires a certain length of time, which is however very variable in different circumstances.—The new Lens gradually acquires a greater degree of solidity and thickness.—Its form depends much on the extent of lesion of the capsule.—The capsule has, in all cases of re-production of the Lens, been observed to retain its transparency.

---

### MULLER ON ENCHONDROMA.

Professor *Muller* was the first who described, under the specific appellation of Enchondroma, a variety of morbid growth, which had previously been classed under the general term of Osteosarcoma, or Osteosteotoma, or cancerous degeneration of the bones, &c.

According to *Muller*, this disease is a fungous tumor of the osseous structure, and of the surrounding soft parts, as of the glands, for example. Its external appearance resembles a good deal that of a common potatoe; it occasionally acquires the size of the closed fist; the skin over it usually remains intact: the tumor is found on dissection to be formed by an agglomeration of small tubercles that are filled with a substance of the nature of soft cartilage. When such a morbid growth is carefully examined, it is observed to be composed of two substances that are obviously distinct from each other; viz. one that is of a fibro-membranous character, and is formed of cells of different sizes; and another that is contained within a fibrous envelope, is of a white colour, and has less consistence than cartilage. Microscopic inspection, however, proves that this inclosed substance exhibits all the features of cartilage; viz. cells of a round or irregularly oblong shape, with granulations within. It not unfrequently happens that the substance of an Enchondromatous tumor is, as Cartilage is in the embryo, altogether cellular in structure. The result of chemical examination confirms that of microscopic inspection; for the presence of *Chondrine* is readily detected in the morbid substance.

Enchondroma presents itself under different aspects. When it affects the soft tissues, it is usually invested with a thin envelope that looks like cellular texture. It does not attack the articular surfaces; and it generally leaves also the muscles and tendons intact. The disease is of much more frequent occurrence in the osseous than in any other tissue: the bones of the metacarpus and of the digital phalanges are especially subject to its development. In some cases it appears as a bony Envelope, the normal osseous network having become softened and being replaced by a mass of Enchondroma; while, in other cases, the morbid growth is without any bony covering, and its surface is then 'bosselée,' and formed by an agglomeration of rounded corpuscles. The first of these forms usually affects the phalanges, and the second the spongy bones of the carpus, &c.

An interesting dissertation, De Encondromate, was published last year at Erlangen by Dr. *Jacob Herz.*—*Gazette Medicale.*

---

### The Insanity of Don Quixote.

It is well known that *Sydenham,* the great physician of the 17th century, recommended the reading of Cervantes' inimitable novel to all his hypochondriacal patients. He might have recommended this marvel of book-writing in a still higher point of view, and for still more important purposes; viz. as teaching, in the most attractive of all ways, that of example, some of the profoundest lessons of Ethics and Philosophy. In his life-like portrait of his hero, Cervantes has left us an admirable picture of one species of Insanity or mental alienation; admirable not less for its scientific accuracy, than for its extraordinary literary excellence. A Spanish physician, Dr. *don Antonio Hernandez Morejou,* has illustrated this point remarkably well in his curious work, entitled, "Beautés de la Medecine Pratique decouvertes dans l'ingenieux Chevalier de la Manche." The following extracts, from M. *Puibusque's* recent work on Spanish and French literature, may prove acceptable to our readers.

"*Cervantes* has read alike the head and heart of man; he is one of those torches of Science which throw light on all the aspects of the human mind; one of those anatomists of thought who have seized and revealed the most hidden secrets of the intellectual organism. The monomania of Don Quixote is not a fictitious and fanciful insanity; it is complete and perfect in all its parts; it is a portrait drawn not by the imagination, but from strict and most accurate observation, having all its traits combined and blended together most faithfully, according to the laws of Nature: in a word, no madness could be more normal."

The most striking features of the Don's insanity, in reference to its exciting causes, are well exhibited in the following table.

"Temperament bilious and melancholic.—Don Quixote was tall, meagre, dry, hairy, of a yellowish complexion, and sombre in his character.

"Age mature; crisis of manhood.—The Don was nearly 50 years of age.

"Culture and fertility of the understanding.—He was full of *esprit,* had an excellent memory, and his acquirements were so various, as to embrace a knowledge of almost every subject.

"Pride of blood and vain glory.—Don Quixote was an *hidalgo*, a descendant in the direct line from Varon de la Alcurnia, of Guttiere Quijada, conqueror of the sons of the Count St. Paul.

"Laborious and violent bodily exercise.—He was a keen sportsman, and especially fond of coursing.

"Sudden transition from activity to inertia.—The Don began to forget, from day to day, the management of his affairs, and even to lose his passion for the chase.

"Diet of heating, viscid, and not very nutritious food.—On *gras* days, he usually lived on cold minced meat, strongly spiced; supped on lentiles every Friday and Saturday; and added a pigeon to the repast on Sundays.

"The summer solstice, and autumnal equinox.—Don Quixote's worst attacks were on the 23rd of July, the 17th of August, and the 3rd of October.

"Amorous feelings.—The Don was ever so much under the influence of the tender passion that he needed not to see his inamorata to adore her, as the very perfection of all that was admirable.

"Excess of reading.—He sold all his property to purchase books of chivalry and romance.

"Long and repeated watchings.—After reading a whole night, by the mere light of the moon, he never thought of taking rest by day; and thus it was that, by reading much and sleeping little, he so scorched and dried his brain that at length he lost his reason."

From the enumeration of the causes, the conscientious doctor passes on to point out the symptoms of the case in these words:

"The term insanity is generic, and comprises several species, each of which again exhibits more or fewer varieties. The symptoms are correlative in their nature, and correspond with the diversity of the causes which produce them. Don Quixote, in losing his judgment, had lost the power of distinguishing falsehood from truth; he was now governed solely and entirely by his imagination, and he believed everything that he had read of in his books of romance; he dreamed of nothing else but adventures of chivalry and protestations of love; all these dreams were regarded by him as so many realities, which could only be pursued by his becoming a knight-errant. Therein consists the special and distinctive character of this strange hallucination; the *ensemble* of the attacks, and of all the circumstances connected with them, constitutes what medical men have called the *Syndrome symptomatologique*. The disease of Don Quixote does not for a moment escape the eye of the diligent observer; even in the apparent disorder of its phenomena, it will be found to follow the necessary course of its nature, whether this be manifested by a transport of fury, by a fantastic impulse of arrogance, by warlike enthusiasm, or by amorous gaiety. We perceive that, in every paroxysm, the external objects which are brought into contact with the senses of the patient, instead of producing normal sensations and regular images, disturb his judgment, and are presented to his imagination only under an aspect that is comformable to the internal disposition of his excited brain."

"When the poor knight has reached the very acme of his madness, at the time when he has determined to remain alone and in complete nudity in the Sierra-Morena, what is the expedient resorted to by his friends to draw him out and bring him home?

"They appeal to and accuse the illusions of his mind by strange and most droll *travestissements*. The curate and barber, disguised in the most ridiculous attire, and the beautiful and unfortunate Dorothea conspire together in the most amusing manner; and every one knows how well the plot succeeded. The latter, falling down at the feet of the Knight, tells him that she is the Princess Micomicona, relates in the most touching mood all her troubles and distresses, and implores him to avenge her griefs. Deceived by this stratagem, the Don leaves his wild retreat, and allows them to bring him along to an inn, where a deep sleep, interrupted only by somnambulism, arrests the progress of his delirium. Soon he falls into a state of profound atony; he is easily mastered, and now we see him carried along to his home in a common cart or waggon, his eyes fixed, his mouth closed, and his head bent down, like a man who has not yet recovered the bewilderment of a frightful dream. Nothing could be more prudent than the resolution of the curate and barber not to visit their friend for a month afterwards, in case their presence might awaken any former associations."

"The moral phenomena, which mark the return of Don Quixote to reason, follow each other in so exact a succession, and are described with such wonderful fidelity that, as Dr. *Morejon* remarks, we might suppose that the Spanish romancer had stolen the pencil of the old man of Cappadocia; and indeed the description given by Cervantes has this great advantage over that of Hippocrates, that it represents to our minds an action and not a mere definition: in place of there being only a cold lifeless object of the intellect, we have exhibited before us a most amusing personage, with whom we may converse; who diverts us with his drollery, and fascinates us with his talk."

---

### On the Traumatic Curvature and Incomplete Fracture of the Long Bones in Childhood.

This subject has hitherto not been examined with the attention which it deserves. M. *Thore*, after alluding to the cases which have been published by MM. *Thierry*, *Villaume*, *Gulliver*, *Mondiere*, &c. relates the particulars of three, which have occurred under his own observation. In the first of these, a child, eight years of age, had the fore-arm bent considerably forwards, in consequence of a fall. In another case, the curvature of the fore-arm was backwards: it occurred in a child three years old, and, from having been neglected, the deformity remained uncured. The third case—some of the details of which we now give—exhibits an instance of a curvature and an incomplete fracture taking place at the same time.

*Case.*—A child, six years and a half old, fell down a stair-case. When visited, the fore-arm was found to be tense, swollen, and very painful on the slightest pressure. The surgeon, that was called, fancied that either the radius or the ulna was fractured: but no *crepitus* could be perceived upon the most attentive examination. The fore-arm was obviously curvated, the concavity being directed forwards; there was also a notable prominence at the posterior and inner side of the limb. By keeping up extension for a few minutes, the curvature was observed to be considerably lessened for the time; but, in consequence of the great pain induced, nothing more could be then done than merely to apply leeches and a lotion.

M. *Thore* did not see his patient again for five months; at which time there was still a slight curvature at the seat of the injury. Moreover, immediately above the middle third of the ulna, and more especially at its inner part, a rounded projection—that was evidently caused by deposited callus—could be distinctly felt. The radius appeared to be quite intact.

Most of the cases of curvature and incomplete fracture have occurred in the fore-arm. Three instances have been observed in the leg, and, in one, the arm or humerus was the bone that was injured.

From a good many experiments, which M. *Thore* has performed on the bones of infants and young children after death, he infers that the tendency to curvature and incomplete fracture is greatest about the second year of life. After the 12th or 14th year, these accidents are of very rare occurrence indeed.

In most cases, the deformity will gradually yield to the application of moderate pressure upon the convexity of the injured bone. Occasionally, indeed, no inconsiderable force has been found necessary to straighten the curvature.—*Archives Generales.*

---

### ON THE MECHANISM OR NATURE OF ATROPHY.

The following extract, from a standard work of medical literature on the Continent, is a tolerably fair sample of the spirit of abstruse and fanciful speculation, if not of the philosophic *clairvoyance*, that characterises so many of the writings of the German physiologists.

"Every act of nutritive crystallisation takes place on the outside of the capillary vessels, in a fluid derived from the blood (*plasma,* vel *cysto-blastema.)* The element of formation is the cellule. This possesses a proper individual life, in virtue of which it is developed (plastic force); moreover, it has the faculty of inducing peculiar chemical changes in the materials which it derives from the *cysto-blastema* by its inner and outer surfaces (metabolic force). Atrophy may be caused, 1, either by the general nutritive fluid ceasing to circulate in the minute vessels, and by the consequent desiccation of the organ; 2, or from the *plasma* being deficient in the formative materials; 3, or from a morbid condition of the cellule itself, rendering it unfit to fulfil its functions; or 4, from some peculiarity in the state of the nervous influence. The cause of atrophy may also reside in the work of decomposition—which consists in the return to a liquid state of the materials that are expelled from the cellule, and absorbed into the torrent of the circulation;—or from an excessive tendency of the organised matter to become liquified; or from a morbid predominance of the excretory process attracting all the organic matter within its reach." Having laid down these principles, Dr. *Caustall* proceeds to examine the internal and external influences, which are apt to induce the state of Atrophy in any part.—*Handworterbuch der Physiologie, art. Atrophie.*

---

### FATAL CASE OF GLANDERS, WITH REMARKS.

A man, 55 years of age, was accidentally wounded in the cheek by one of the upper-jaw teeth of a glandered horse, while he was attempting to give the animal drink. The wound bled a good deal at the time; it was bathed first with salt and water, and then with flowers steeped in brandy. He remained quite well until the next afternoon, when he was seized with shivering and general *malaise.* He was hot and restless during this (the second) night and complained much of headache, pains in different parts of the body, more especially in the seat of the wound. The face became affected with Erysipelas; the edges of the wound looked of a blueish cast, and were covered with phlyctenæ; there was exceeding prostration of strength and spirits; the breathing was hurried and oppressed, and the pulse very rapid and small. The pains in the joints, especially in the left hip, right knee, and lower part of the leg on this side, immediately above the ankle, were exceedingly severe.

For two or three days, the symptoms appeared to become mitigated under the treatment that was adopted; but on the seventh day we read that, in addition to the symptoms already enumerated, there was a sanious discharge from the right nostril, and petechial spots scattered over the surface of the body. The patient, who was every now and then delirious, frequently complained of intense pain in the left hip, the integuments over which were swollen and œdematous. On the 13th day, the following is the report of the symptoms: dorsal decubitus, extreme prostration, profound stupor alternating with delirium, severe headache, eyelids œdematous and of a livid hue, the conjunctivæ deeply injected, incipient opacity of the corneæ; wound suppurating freely; the malar bone exposed; discharge of bloody matter from the nostrils; the nasal mucous membrane red, sprinkled over with sanguineous crusts, but without any visible ulcerated spots; lips sooty; tongue dry, yellowish and filthy; intolerable thirst; difficult deglutition; numerous brown papulæ, surrounded with red areolæ, over every part of the face, and confluent on the nose and lower eyelids; enormous "empatement," but without any sense of fluctuation of the left hip; distinct fluctuation immediately above the right ankle; a variolous-looking eruption on the abdomen and extremities, with phlyctenous and livid spots between the pustules. The patient died next evening.

*Dissection.*—Passing over the description of the wound itself and of the cutaneous eruption, we may notice that the abscess above the right ankle was found to be situated under the fascia of the leg, and extended inwards into the substance of the muscles. The nasal mucous membrane was of a very dark colour, much softened in texture, and infiltrated with purulent matter: here and there were pustules and ecchymosed spots, especially on the surface of the spongy bones. The tonsils and the pillars of the fauces were bedewed with pus. The epiglottis was œdematous, and speckled over, at one part, with minute sanguinolent spots.

The lining membrane of the larynx and trachea very highly congested, and dotted over with numerous whitish papulæ, like the incipient cutaneous pustules. Cellular texture underneath the sternum and pleura emphysematous; considerable serous effusion within the left pleura; lungs containing numerous subpleural purulent nuclei, which varied in size from that of a pea to that of a pigeon's egg, disseminated through their substance. The blood within the heart and large vessels was very black; but there were no coagula.

The reporter made some experiments on asses with the purulent discharge of this patient. Some of the pus from the nostrils and from the abscess near the right ankle was taken on the day preceding the patient's death, and inserted, by pretty deep scarifications, into the flesh about the shoulders of a healthy and vigorous ass. The animal speedily exhibited all the symptoms of acute glanders, and died on the seventh day after the experiment.

The post-mortem appearances were in every respect such as are usually found after fatal cases of the idiopathic disease.

M. *Landouzy* quotes with approbation the remark of M. *Bouley*, that 'Acute Glanders is a highly contagious disease; contagious by the product of the nasal secretion; contagious by the expired air; contagious by the blood; and contagious by the tissues of the dead body. After the fever of incubation, when the virulent eruption takes place, the infected animal exsudes, so to speak, the morbific matter from every pore.' The observations of MM. *Rayer*, *Breschet*, and others, have most satisfactorily shewed that the disease is transmissible to other animals besides those of the solipedous family; for example, to dogs, sheep, and goats. A recent melancholy case at the Hôpital Necker clearly establishes the fact that it may be conveyed from one human subject to another; one of the *internes* at that hospital having died of the disease, caught by examining the body of an ostler who had fallen a sacrifice to it.

It appears that in the three years from 1837 to 1840, no fewer than 27 persons have died in Paris of the glanders. M. *Landouzy* condemns the too common practice in the French metropolis, of feeding dogs and sheep with the flesh of horses that have died of the disease.

"As to any essential difference between the acute and chronic forms of Glanders, we do not believe," says this gentleman, "that such exists; and the adoption of the contrary opinion has, on more than one occasion, led to a fatal security. We know of many indubitable cases of Farcy communicated from horse to man, and *vice versâ*. If the identity of these two affections, and if the circumstance of their passing from the acute to the chronic state, and from the chronic to the acute, warrant certain scientific distinctions, they will not permit us, without great rashness, to establish any legal distinction."—*Gazette Medicale.*

---

### M. Brachet's Opinion of Animal Magnetism.

M. *Brachet* may be justly considered a high authority on all topics connected with the phenomena of innervation, in the state alike of disease and of health. His great experience and clearsighted practical sagacity entitle his opinions to universal respect. As a matter of course, he, like other medical men of experience, has seen something, and read more, of the vaunted marvels of Mesmerism, as it has been revived of late years by *Dupotet*, *Elliotson* and others. Let us hear what judgment he has formed on the subject.

"From the Magnetism of Mesmer has arisen that other jugglery, denominated Animal Magnetism. Twenty times beaten down by science, and reason and facts, every now and then it has again lifted up its head, more ridiculous and amusing, indeed, than dangerous. We do not however mean to deny the effects which may be induced in persons of highly nervous constitutions by the *passes* and other grimaces that are usually practised. In the magnetic stupor of the animal energies that is sometimes induced, the entire nervous system is compromised; and this influence may unquestionably appease pain and spasmodic contractions for a time, by acting powerfully on the imagination. We can readily conceive the possibility of this; and certainly there is no lack of cases of alleged cure in hypochondriacal, as well as in many other, ailments. Although we have heard of such, we have not ourselves met with any well-authenticated examples. In our opinion, this Animal Magnetism, even when divested of all the apparatus of Charlataneiy, is on the whole more likely to do harm than good in the disease now under consideration (Hypochondriasis). If such be our opinion of Magnetism, we need scarcely say that we equally discredit all the recorded wonders of Somnambulism, the exhibitions of which are now almost entirely limited to rogues, whose only object is to attract the public notice, and rob their silly dupes.* These distant voyages without moving from off one's chair, these divinations, these transpositions of the senses, &c. are only so many clever tricks contrived to amuse the weak and entrap the foolish. It may so happen that a poor silly hypochondriac, who is strongly prepossessed in favour of this culpable jugglery, appears for a time to derive some benefit to his health; but then it is only from his becoming the dupe of his credulous fancy, and not from any direct or actual sanative influence bestowed."

We observed in a recent number of the Medical Gazette a quotation to the same effect, of the opinions of the celebrated *Müller* of Berlin, on the subject of Animal Magnetism. How long will any men of education allow themselves to be imposed upon by the juggling tricks of clever rogues, and the paid-for testi-

---

* Within the last few weeks, the mountebank mummery of MM. *Marcillet* and *Alexis*, who were fleecing the West-end ignoramuses at the rate of five guineas for every private *seance*, has been covered with the ridicule and contempt which it deserves, and these knaves have been *chassés* from the metropolis, in consequence of the clever exposure of their lying and dishonest tricks.

mony of credulous women? Medical men, at all events, should know better; for they must have studied the history of the nervous system and its functions only indifferently well, not to be aware that many startling, and not easily explicable, phenomena are apt to occur during the progress of some of the Neuroses.

---

### Remarks on the Molluscum Contagiosum.

The *Molluscum Contagiosum* of Bateman is a disease of very rare occurrence in France. *Biett* has never seen an example of it; but Professor *Andrieu* has recently published two cases of it.

A girl, eight years of age, had a number of *verrucæ* on the chest, neck, and lower half of the face: she had, it was supposed, contracted them at a boarding-school, where she was much in the company of another girl who was similarly affected. This dermatosis exhibited the following characters, when first examined by M. *Andrieu*. "In what might be called the first stage, each tubercle was of a conical shape, shining on the surface, and of a paler colour than the surrounding integuments. The apex of the tubercle was truncated, and in its centre there was a grey or blackish point, giving it an umbilicated appearance: the basis was large and sessile.

"In the second stage or period, the tubercle had increased in volume; its basis was now pediculated; its free part was round and distended; and the umbilicus or central spot was more prominent. By degrees, the little tumor assumed a red hue, and became apparently the seat of a peculiar kind of inflammatory process. Upon being compressed, there escaped from the central opening a small quantity of cheesy-looking matter, very similar to what is squeezed out of the cutaneous follicles in *Acne*.

"In the third stage, these warty-looking tubercles resemble, in a very striking manner, gooseberry grains of a medium size. Reddish at first, subsequently of a dull red hue, and at length of a blackish colour during a process of destructive maturation, they end by becoming completely desiccated, and then fall off, leaving behind them a discolouration of the skin, somewhat analogous to that which is left from the vaccine inoculation."

Two months after his first visit, M. *Andrieu* again saw his patient, and he then found that her mother had become affected with the disease. Tubercles, similar to those which we have now described, had made their appearance on her face and neck, and afterwards on her breast and fore-arms.

Frictions with a pommade—composed of four parts of arsenious acid and thirty-four parts of lard—caused the complete dispersion of the tubercles in both patients: the time required for the cure being about three months in the case of the child, and four in that of her mother. No constitutional remedies were administered in either instance.—*Gaz. Med. de Montpelier.*

---

### Actual Cautery in certain Uterine Diseases.

It is under the inspiration of M. *Jobert* that this memoir (by a M. *Lauges*) has been written. For several years past, the surgeon of the St. Louis Hospital has occasionally substituted, with very remarkable success, the use of the actual cautery in place of the various potential caustics in the treatment of certain uterine diseases.

The cauterisation is effected by means of an iron or steel rod, passed through a speculum of ivory—a material that is a bad conductor of heat. The instrument should be heated to whiteness; for in this state it is less apt to adhere to the part to which it is applied—a result that might be apt to detach and bring away the newly-formed eschar.

If the vagina be not touched with the cautery, this operation causes little or no pain, and it is rarely followed by any severe re-action; the process of menstruation is not sensibly disturbed, however near to a catamenial period the application of the cautery may have been made.

As to the indications for the use of this remedy, it is particularly well suited—

1. To deep, exuberant, fungous ulcerations of the os and cervix uteri, complicated with hæmorrhage, simple hypertrophy, or engorgement with ramollissement or induration.

2. To hypertrophy with uterine Catarrh, but without ulceration.

3. To the ulcerations that often attend a softening of the tissue of the cervix uteri, when this is apt to bleed upon the slightest pressure.

4. To rebellious neuralgia of the Cervix.

5. To unhealthy ulcerations, for the cure of which excision of the cervix uteri has been recommended and performed.

M. *Jobert* has triumphed with the actual cautery over an affection, for which the patient had twice undergone this formidable operation.—*Journal de Chirurgie.*

*Remark.*—We need scarcely say that our object in publishing these observations is not to recommend the practice here enjoined, but only to record what is going on among our brethren on the Continent. We trust that the day will never come when any rational surgeon in England will propose to apply the actual cautery to the uterus for the cure of neuralgia of its cervix!

---

### M. FORGET ON DISEASES OF THE HEART.

"No one before the time of *Laennec* ever thought of questioning the accuracy of the old doctrine—sanctioned too by the imposing authority of *Bichat*—that the sounds of the heart are owing to its impulsion or stroke against the ribs and sternum. We have no intention at present of canvassing the merits of the various theories that have been proposed on this subject of auscultatory investigation during the last twenty years. Suffice it to say that, in our opinion, the theory, that refers the cardiac sounds to the action of the auriculo-ventricular and aortic valves, appears the most probable; although we are quite ready to admit that other influences may have something to do with the phenomena in question. Whatever view we adopt, this one fact is beyond dispute, viz. that whenever the valves become altered or diseased, a sensible change in the tictac sounds of the heart is invariably to be perceived."

"From their community of origin, we may infer that it is not possible, in a practical point of view, to discuss separately, and apart from each other, the subjects of valvular contraction and valvular insufficiency; and therefore that it is much more logical and more conformable to nature, to take for the basis of our clinical enquiries valvular alterations in general, as constituting the fundamental element of organic diseases of the heart.

"Now these alterations are, in an immense majority of instances, the starting-point and direct cause of cardiac dilatation and hypertrophe.

"Of 29 cases of valvular disease, examined by dissection, we found that in 9 the aortic valves alone were affected; in 10 the mitral valve only was the seat of morbid change; and in the remaining 10 both these valves were diseased. On one occasion only have we ever seen the tricuspid valve affected; the pulmonic valves never.

"We may therefore fairly conclude that the lesions of the cardiac orifices are, in very many cases, multiple; and that, as these lesions give rise to very analogous symptoms, whatever be the orifice that is affected, the diagnosis of the exact seat of the alteration is often very difficult, if at all possible, during life: in practice it is fortunately not of much importance to diagnosticate the precise nature and seat of the valvular disease.

"The essential point to determine is, whether there be such a disease existing, or not. If there be, we may rest assured that it is in the left or systemic cavities; but whether it be the aortic or the mitral valve that is affected, it is never easy to say. There has been no little parade of scientific and technical discrimination very needlessly expended in attempting to point out the diagnostic symptoms of different cardiac diseases. Be it remembered that the aortic valve is not distant from the mitral more than by a ring of a few lines in breadth. How then can we believe that their diseases will be marked by any very distinguishing symptoms?"

"Considerable difference of opinion has existed as to the explanation to be given of those instances of confirmed valvular disease, in which the usual, and, we may almost call them, the characteristic *bruits* are absent. Making allowance for those cases in which the alleged absence of the auscultatory signs may be fairly attributed either to the inexperience or the want of tact of the practitioner, we can have no difficulty in mentioning certain peculiarities which will sufficiently account for the occasional indistinctness of the abnormal sounds in valvular disease of the heart. For example, the pulmonary murmur may be so loud, as more or less completely to mask the cardiac *bruits;* in the same way as the interposition of a portion of lung between the heart and ribs may deprive the cardiac region of its usual dulness on percussion. Another cause may be the abnormal degree or amount of force in the heart's contractions. If, for example, this force be excessive, there is almost necessarily such a tumultuous confusion present, that the characteristic sounds are quite 'denaturés;' and, on the other hand, if it be deficient, the sounds are scarcely, if at all, produced;—for the very simple reason that the valves cease to vibrate, in the same manner as the cords of a violin remain quiescent, if too lightly touched with the bow. This is precisely what is often observed in the last stage of cardiac disease, when the heart beats with extreme debility."

"Another argument, urged by the Vitalists against the mechanical theory of the sounds of the heart's action, is derived from the occurrence of certain cases, in which, it is acknowledged, the existing sounds or *bruits* do not exactly correspond with the character of the existing lesions. Now it is quite true that it is often very difficult to explain altogether satisfactorily the various circumstances which cause the cardiac sounds to vary their character from day to day. What we have said above will partly serve to solve this difficulty; for, just as the excess of force or feebleness in the contracting power of the ventricular parietes will account for the non-production of the special sounds, may we not presume that the same causes will explain the irregularity of their relative strength and weakness under certain conditions of the circulation? Suppose, for example, that, in a case of contraction with insufficiency of the aortic valves, the left ventricle can contract only imperfectly, while there is no impediment to its free dilatation, then the first sound will probably be absent, and the second one only will be perceived. Moreover, let it be remarked that, if any ambiguous case be attentively watched for some time, the characteristic auscultatory signs of the existing lesion will generally be recognised; for the peculiar sound, that has been wanting to-day, may have been heard yesterday, and will be so again to-morrow. The actual disposition of the altered orifice, its degree of tension, its particular conformation, &c. should all be taken into account, in our enquiries upon this subject; for we well know that a slackened cord does not vibrate like one that is tense. The Humorists may still invoke the crasis of the blood, and the Vitalists the contractility of the vessels, to account for some of the acoustic phenomena of the heart's action; but, even admitting such interpretations, are not these conditions really and truly *physical* circumstances? We do not assert that valvular thickening is the only and the exclusive cause of abnormal cardiac sounds; but this we *do* assert, that it is by far the most common cause of them; and, whatever difficulty we may occasionally find in explaining certain phenomena, it is better to charge it to the insufficiency of our means of exploration than to the inconsequence of Nature's acts and operations: *physica physicè explicanda.*" —*Gazette Medicale.*

---

### MATERIALISM OF MODERN PHYSIOLOGY.

M. *Virey* has been a prolific author in his day. Most of his writings are of a physico-moral character, having for their object the exposition and illustration of the reciprocal influences of mind and matter, throughout the wide and varied field of animal existence. With the exception of his "Natural History of Foods, Medicines, and Poisons," and his "Treatise on Pharmacy"—which has passed through several editions—all his works belong to that mixed category in which the natural sciences fraternise with philosophy—the embrace, however, being sometimes so intimate and close between them that the latter is fairly overlaid by the former.

So far back as 1809, he published a work entitled "Art de Perfectionner l'Homme," in which he discussed the character, manners and complexion of celebrated men, the peculiarities of the female organisation, and the incessant and often stormy action and re-action of moral and physical influences on the human frame in general. He subsequently wrote a separate volume on "Woman considered in a physiological, moral, and literary point of view." In 1817 he brought out his "Medico-philosophical Researches on the Nature and Faculties of Man;" and, in a following year, a work on the "Natural History of the Human Race." His recently published "Physiology in its relations with Philosophy," belongs to the same category as several of the preceding productions of his pen. It contains a vast deal of speculation, more fanciful than solid, and more ingenious than convincing, on the difficult questions of vitality, the mutual influence of mind and matter, the relations of thought and feeling with instinct and spontaneous action, the polarity, as he calls it, of the nervous system, or the opposition of the cerebral with the generative pole in vertebrated animals, the relation between the volume of the cerebral mass and the development of the mental faculties, the curious subjects of dreams, hypochondriasis, and madness, &c. &c. Many of his views remind us of the visionary speculations of a transcendental German writer; they often savour far more of the metaphysician than of the physiologist; and unfortunately, if not designedly imbued with the spirit of Materialism, will infallibly be laid hold of by the sceptic in support of his irreverent conclusions. He assigns to the Ganglionic system the seat of the passions; the intellectual powers, he refers to the Encephalon; and he regards the eighth pair of nerves as the bond of communication between them.

The unequal distribution of sensory power, in virtue of the laws of antagonism of different parts, is a favourite dogma with our author; and every now and then he brings it into prominent relief. Thus, we are told that (the anterior and the posterior regions of the body being in an inverse ratio of development) 'every *deploiment* of the posterior muscles or of their actions, as in the case of dancers, necessarily implies the inferiority of the encephalon; a fact that is shewn in the case of leaping and running animals, all of which have a comparatively small head and very little *esprit*.' Poor comfort for *Fanny Elsler* and *Cerito*;—who, in spite of all their grace and spirituality, are physiologically, and therefore must of a necessity be, fools, hopeless fools!

M. *Virey* regards the nervous as identical with the Electrical power. "The functions," says he, "of the nervous system, thus connected with universal life, correspond with the revolutions of the planetary globe, in which the organisms are developed. It is probable that the vegetable and animal kingdoms are simultaneously animated by a biotic principle, the most active element of our world, compared to a subtle fire which seems to be the electric fluid modified into the galvanic and voltaic conditions, according to the mineral, vegetable, or animal fibres which it traverses." Unmeaning verbiage!

Our author agrees with *Lamarck* and *Decandolle* in ascribing to plants the possession of certain instinctive impulsions and acts of spontaneity; but which, in the absence of a nervous system, do not amount to perception.

With the ganglionic system appears the development of instinct in the Invertebrata; and with the cerebro-spinal axis that of intellect in the Vertebrata. The Encephalon is the laboratory of thought, having for its object the perception of the beautiful and true; the ganglionic apparatus is the seat of the feelings, affections and desires, and its end is the useful:—such are the two distinct and antagonist sources of the faculties. The innate ideas are simple original aptitudes, proceeding from the instinct, which is the predominant and pre-existent moving power of animals. "Whether we admit," says our speculative author, "an infinite individuality of instinct in each structure, or whether we recognise a mighty framework of actions foreseen and associated together, every species of which is but a wheel, so to speak, that serves either to assist or to retard each other's movements, there evidently exists a primitive design, by virtue of which every created thing moves in an appointed orbit, as the stars do in the firmament of heaven."

M. *Virey*, notwithstanding such dreamy and bewildering speculations as these, enters his protest against any charge of Pantheism being laid at his door. But, after having placed the origin of the Instinct and Intellect in organised matter, will the mere admission of certain pure and immaterial agencies suffice to clear him from this imputation? Such an act reminds one of the present condition of medical and philosophic writers in Italy, who are obliged to affix, as a heading to their works, an act of Catholic faith, and a disavowal of all heterodox interpretations which may at any time be drawn from their opinions.

The closing words of the writer, from whom we have taken this short account of M. *Virey's* new work, are rather remarkable,—perhaps some will add, pharisaical and unjust: "The anatomist ends in the Phrenology-, the clinician in the Vitalism-, and the naturalist in the Electricity-God. As for us, reader—you may perhaps smile at our avowal—we prefer the Bible and the Gospel."—*Gazette Medicale.*

---

### Greatness of Sir C. Bell's Discoveries; Relation of the Cranium to the Encephalon, &c.

The following generous acknowledgement of the pre-eminent value of the English physiologist's discoveries, we observed in the opening paragraphs of a French review of M. *Foville's* new work—Traité complet de l'Anatomie, Physiologie, et Pathologie du Systeme Nerveux—in the same Journal from which we took the preceding notice.

After alluding to the numerous and unsatisfactory attempts, that have been made in almost every age of our science, to explain the complex machinery of the nervous system, he remarks:—

"Amidst so many hypotheses, there is one idea that stands firm and upright. This is the great conception of *Charles Bell* as to the special attributes of the anterior and posterior roots of the spinal nerves. Based on the most legitimately demonstrative examinations and researches, this discovery still enlightens and directs the entire physiology of the nervous system. Thanks to it, an epoch of positivism in doctrine, and of exactitude in arguments, has replaced centuries of vague explanations and fanciful conjectures. Alone, it has sufficed for the reputation of many men. Since the illustrious discoverer first made known his novel views, one has repeated his experiments, without making any change in them; another has made an application of them under different circumstances; a third has experimented on those animals which approach nearest to man in their organization; and a fourth has confirmed the truth of the principle, that had been proclaimed, by pathological observations;—and all have found in these second-hand discoveries and investigations a very satisfactory reward of praise and reputation."

The writer proceeds to remark that, however beautiful the theory of *Bell* is, it is to be remembered that, if considered as an exposition of the nervous system, it is imperfect and insufficient; for it explains but one part only of this system, viz. the nerves and the spinal marrow; and remains altogether dumb as to the functions of the great mass of the encephalon. The edifice is not completed; it is but part of the scaffolding and structure that has been reared; but these—thanks to the English physiologist's skill,—have been firmly and surely fixed, and are not liable to be again displaced. To finish, on the same plan, a work so successfully commenced, much remained to be done. It was necessary to follow in the encephalon the anterior and posterior fasciculi of the spinal marrow, to point out their course, their irradiations, their terminations, and their mode of connexion and union—whether of the fibres of the same kind among each other, or of the motor with the sensory fibres. This is the labour which M. *Foville* has undertaken, and to the due prosecution of which he has devoted his best exertions for a long series of successive years. As a matter of course, we have no intention of entering upon any anatomical details at present. * * * * *

M. *Foville* has not omitted to examine with due care the subject of the relations between the configuration of the Cranium and the form of the different regions of the Brain. He does not deny that, in a general manner, the volume of its contents is indicated on the cranium by the modifications of its surface; but he qualifies this admission by expressing his belief that, if we wish to remain in the truth, the science, which professes to study these connexions, ought not to attempt more than merely to point out the most general relations, and avoid entering into details that are altogether conjectural, if not positively erroneous. Applying these principles to his own inquiries, he endeavours to determine the causes which preside over the formation of the constant osseous protuberances on the cranial surface. In his opinion, these *bosses* are attributable to the form and development of the cerebral ventricles. It is certainly well deserving of notice that each of these protuberances on the surface of the cranium corresponds in its shape and situation with an inflated or projecting part of the cavities in question. Thus, if we divide the two frontal protuberances by a section perpendicular to their surface, this section, if prolonged through the substance of the cerebrum, will be found to open the lateral ventricles at their anterior cornua. Again, if the parietal protuberance be treated in the same manner, you will come upon the ventricles where they are widest and project most outwardly; and the two superior occipital *bosses* are placed exactly over the posterior extremities of the ventricles.

It is very natural that the development of the brain should thus act on its osseous case. We know that, in the embryo, the place of the cranium is occupied by a membranous pouch which, although simple in some points, is reinforced in others by a fibrous doubling or lining. Now, an organ inclosed in this sac and gradually increasing in volume, will necessarily force outwardly the surrounding walls, and protrude them most forcibly at those points where they are thinnest, and where, consequently, there is the least resistence: hence the projections or protuberances on the external surface of the cranial bones. Various pathological phenomena may be adduced in support of this law; thus, for example, we observe in hydrocephalous patients that all the cranial protuberances increase in proportion to the distention of the cerebral ventricles. In order that this take place, it is necessary that the containing envelope oppose a certain degree of resistance, and that it be closed at all points. Thus the protuberances exist when the cranium contains nothing but water in place of cerebral matter; but they are wanting if, with the absence of the encephalon, there be an opening of the cranium.

---

### Extraordinary Operation; Excision of Portion of the Colon; Character of French Surgery.

The following somewhat marvellous case, which occurred eleven years ago, was only recently communicated by the operator to the French Academy. A man, 28 years of age, had for several years suffered from frequent attacks of severe lancinating pains in the left side of the hypogastric region, which had become much aggravated for about six months before he consulted M. *Reybard* of Lyons. At this time, the abdomen was enormously enlarged from tympanitic distention. In the left iliac region, a hard tumor, as big as an ordinary sized apple, could be felt: it was deep-seated, but moveable. The bowels were very costive; and the patient was much distressed with tenesmus, which usually induced a discharge of purulent matter, tinged with blood, from the anus. No tumor could be felt *per rectum.* On one occasion, a considerable suppuration must have taken place; for, after passing a good deal of matter with the stools, the patient was very sensibly relieved. The opinion, which M. *Reybard* formed of the case was, that a malignant tumor had become developed in the sigmoid flexure of the colon; and, as there was no rational hope of relieving the patient—whose health was rapidly deteriorating—by any internal medicine or external application, he decided on the removal of the tumor by operation. He proceeded in the following manner. Having made a free incision through the abdominal parietes, and a smaller one through the peritoneum, the diseased mass was with difficulty drawn out; and a pretty considerable extent of the mesocolon was embraced between two ligatures, in order to prevent hæmorrhage. A portion of intestine, about three inches in length, was removed with the bistoury, and the mesocolon was cut with scissors.* Several arteries were tied, and the ligatures left long, in order that they might be introduced into the cavity of the gut. The two divided ends of the intestinal tube were then brought together and secured by the overcast suture. When this was duly done, the gut was replaced within the abdomen, and the outer wound was united with three stitches. Things went on favourably until the fifth day, when symptoms of irritation set in; but these were speedily dissipated by the use of leeches and emollient enemata. There was no alvine evacuation until the tenth day, when the ligatures of the outer stitches were removed. Five weeks after the operation, the patient eat solid food, went naturally to the closet, and seemed to be entirely cured. It was not for six

---

* M. *Reybard* described to the Committee, appointed by the French Academy to report upon the case, the tumor as follows: "its size was that of an ordinary rennet apple; it was hard and of a greyish-white colour; it presented to the touch several tubercles; it occupied the two posterior thirds of the intestine—which, when opened longitudinally in front, was found to be considerably contracted." *M. Reybard had lost this interesting pathological specimen!*

months that he began to experience any sharp flying pains in the left iliac region. These gradually increased in severity; and, at length, it was discovered that a new tumor had formed in the site of the old one. The patient's state speedily became worse and worse; his strength failed; and he died, about ten months and a half after the date of the operation. *There was no post-mortem examination!*

*Remarks.*—There are some circumstances connected with this case that must naturally make us pause before we can receive the report with entire confidence. How comes it that M. *Reybard* has allowed so long an interval to elapse between his extraordinary success and any communication of it to the public? How comes it that he did not most carefully preserve the specimen of excised bowel—the only palpable record of his operation? How comes it that there was no post-mortem examination of the parts? It is nevertheless fair to say that the Commissioners appointed to examine and report upon M. *Reybard's* statements expressly state that, "whatever doubts may be entertained as to the nature of the removed tumor, we cannot doubt the excision of two or three inches of the intestine, so precise and circumstantial is the description of the operation that was performed."

This seems to us to be a not unseasonable opportunity of introducing a few remarks on the character of the practice of some of the leading hospital surgeons in Paris. They occur, we may observe, in the course of a statement put forth by M. *Guerin's* friends to vindicate him from the charges that have been made against his honour, for refusing to submit all his hospital reports to the Commissioners that were lately named by the Academy to report upon his tenotomic operations.

"What would MM. *Roux, Velpeau,* and *Gerdy* say, if any member of the Academy proposed to subject to a Committee of Enquiry the results of the practice, proverbially so unfortunate, of the first; or the ablations, so readily repeated, of cancerous tumors by the second; or the disastrous results of the attempts made by the last-named gentlemen to effect a radical cure of hernia? Let these gentlemen be required to give up all their documents and hospital reports, under pain of being accused—doubtless, very unjustly—the one of want of skill, the other of rashness, and the third of all that might emanate from certain papers which, it is said, he preserves with so paternal a solicitude. Why not apply to them the *lex talionis,* and tell them that the enquiry proposed is all for the benefit of science and humanity? Surely what is right in one case, cannot be wrong in another of exactly analogous import. But it always so happens that those very persons, who are most prying into the affairs of others, are ever the most jealous of their own being submitted to public gaze. One might suppose, as M. *Velpeau* has pretended, that to a certain point the interests of science should level all personal differences; but, alas! this is not possible, when we find ourselves so situated that the spirit of party takes the place of science, and when passion usurps the seat of calm judgment. As to science, it is pretty clearly seen that its interests are but little thought of in the attacks of M. *Malgaigne;* and M. *Guerin* has most fairly recalled to the minds of all who take an interest in the controversy, that the hospital Committee, composed of distinguished members of the Academies of Medicine and of Science, is now engaged in the examination of the subject under dispute. If the regard for the promotion of science were indeed the only principle that actuated the opponents of rachidian myotomy, they would not merely wait with patience for the report of this Commission, but they would of themselves remember that M. *Guerin,* eight or ten months ago, applied to the Academy to appoint a commission to which he would willingly submit all his patients, and the reports of all his operations. But the question now is, not so much about the interests of science or of truth, as about personal enmities, indecent rivalries, and professional jealousies, which prefer a calumnious attack upon private and public character to the discussion of theoretical and practical controversies."

So much for the angry bickerings between many of the members of our profession in the French metropolis. The much-vaunted Royal Academy of Medicine, while doubtless it serves to keep up an active spirit of Enquiry and Communication, is, alas! too often made the channel not only of much empty *niaiserie*, but also of much angry controversy, and occasionally, too, of most unbecoming reproaches.

---

### Another Case of Artificial Anus in the Loins.

We have, on more than one occasion, directed our readers' attention to the bold, and, we think, most hazardous practice of M. *Amussat*, in cases of diseased rectum and other maladies accompanied with obstinate and unyielding Constipation. The question will naturally occur to many, was there a fair trial made of all the ordinary remedies and means of relief, before resort was had to the formidable step of opening the bowel in the lumbar region? The last case operated upon is the following:—

A woman, 53 years of age, and hitherto of a robust constitution, began to be annoyed last April with a gradually increasing constipation of the bowels, and occasional attacks of sharp colicky pain. These attacks became more frequent and severe, and the fæcal matters, evacuated after the use of enemata, were found to be small, flattened, and smeared with a sanguinolent mucus. From the 12th of June, there was no intestinal evacuation either of fæces or of wind; and all means, that were tried to cause the bowels to act, utterly failed. No obstacle could be detected by an examination *per rectum*. On the 1st of July, M. *Amussat* performed the operation of making an opening into the descending colon in the left lumbar region, without dividing the peritoneum. After exposing the intestine by an incision carried through the integuments and muscles in this part, he passed a tenaculum into it, in order to secure it in its position, and then made a vertical opening in it with a pair of scissors. When this opening was sufficiently enlarged, the cut edges of the gut were fixed to the wound in the integuments, at the anterior angle of the wound, by means of three stitches. Injections of tepid water facilitated the evacuation of the fæcal matters.

The result was most satisfactory; no inflammatory or febrile re-action supervened; and, by the 30th of the month, the patient was "dans l'etat le plus satisfaisant." The artificial anus perfectly fulfilled its functions, the bowels acting very regularly; during the intervals between each evacuation, the wound was kept closed by a wax bougie.

---

### M. Trousseau on the Signs of Auscultation in Young Children.

Every experienced physician must have found—if he has taken the trouble to examine the subject—that auscultation is of comparatively little value in the diagnosis of pulmonic diseases in early life. It is not often that the young patient can be kept sufficiently quiet to enable us to make the proper examination; and, moreover, the respiratory murmur is usually so loud and boisterous—especially upon any excitement—as completely to overpower any abnormal *bruits* that may be present. Fortunately the practitioner does not often feel the need of any extraneous means to aid his diagnosis in the thoracic diseases of infancy; the rational symptoms, as they have been rather absurdly called, being usually quite sufficient. The following remarks were made by M. *Trousseau* in his clinical lectures at the Hôpital Necker.

"If the child be perfectly quiet, the breathing does not sensibly differ from that in the adult; the inspiration is rather noisy, while the expiration is scarcely perceptible; moreover, the former is exceedingly active and slow, while the second, on the contrary, is rapid and purely passive. But, if the child be restless, the inspiration is immediately rapid, and the expansion of the lungs cannot be perceived, while the expiration is, at the same time, slow, and accomplished with the aid of all those muscles which usually concur to the performance of this act. The air issues from the glottis in a small noisy stream. The expiration, therefore, is here essentially active, the very contrary of what it is in the normal state: moreover—and I insist upon this point—it must be very slow, while the act of inspiration is performed rapidly.

"This new rhythm it is very necessary to be aware of, because the character of certain pathological auscultatory sounds, which are thence derived, is more or less altered in consequence. In truth, if, as M. *Beau* believes—and in this opinion I quite agree with him—the blowing sounds and their numerous modifications really take place and are formed in the larynx, and are transmitted to the ear through the indurated lung by the air in the trachea and bronchi, it must follow that they (the sounds) will be the more distinct and obvious in proportion as the passage of the air is accompanied with the most blowing noise in the larynx. Now this is what we perceive to be the case in the adult. The inspiration is slow, and the expiration is rapid; the inspired air therefore passes more silently through the larynx than that which is expired; and thus it is that the blowing sounds are most distinctly audible during the act of expiration. But if—as in the case of the restless child—the inspiration be performed rapidly instead of slowly, the blowing sounds are heard during expiration when it (the child) is calm, and during inspiration when it is restless and crying."

---

### REMARKS ON HYSTERICAL PARALYSIS.

This form of the Protean disease has not attracted so much notice as we might have expected. Authors have seldom described it in a very distinct or special manner. All that *Georget*, one of the most recent writers, says respecting it, is in these words: "The troublesome consequences of hysteria are convulsive neuralgias, spasmodic retractions of various parts, paroxysms of suffocation, partial palsies, usually incomplete, of the senses and voluntary movements, &c."

M. *Piorry*, in his clinical lectures at La Pitié Hospital, has occasionally described some of its forms under the appellation of Hysteric Anervia. As in paralysis from the action of lead upon the system, there is no appreciable lesion of the nerves; but beyond this the analogy between the two forms of the disease; for the one continues until the cause be removed; while the other goes and comes without any evident cause, being so volant and capricious as often to defy all attempts to pursue it. All that we can say respecting it is, that it very generally follows upon any violent shock or exhaustion of the nervous system: an atony, inertia, and, lastly, a palsy is the result. When this atony affects the brain, there is a loss of intellect, a palsy of the mental faculties; and when the nerves are affected, then we have a palsy of those parts which the nerves supply; and this state continues until the loss of nervous power be restored, and the system recovers its former strength and repose.

The hysterical, like the other forms of paralysis, affects sometimes the contractility of the muscles; at other times the sensibility of the nerves; and in a third set of cases, both these systems suffer together and in an equal degree: the last-named variety is the least frequent in hysterical habits.

The phenomena of Hysterical Palsy are always more or less erratic; they disappear with facility, again to make their appearance ere long. Sometimes one hysterical phenomenon is replaced by another of a different sort. Thus *anæsthesia* may be replaced by *amyosthenia* or loss of muscular contractility, deafness by blindness, and so-forth. Their duration varies from a few minutes to several days or weeks; nay, they have been known to last for months, and even years, and then quickly and suddenly cease, with as little warning as when they came on. A woman, while the catamenia were upon her, dreamed that she addressed some words to a man, who did not answer her: it proved that he was dumb. The effect of this surprize was that, on awaking, she found that she had lost her speech herself. By the use of venæsection, and of a warm bath, she recovered her speech in the course of two or three days.

Perhaps Amaurosis is one of the most frequent forms of hysterical Paralysis of the nerves of special sense; deafness comes next; and then the loss of smell and taste. According to the observations of M. *Gendrin*, the loss of smell is a not unfrequent form of Saturnine Paralysis. Dr. *Gabrini* relates a curious case of a woman who, upon the cessation of the menstrual process, became affected first with loss of speech; then with palsy of the extensor muscles of the right leg, accompanied with anæthesia of the surface of the thigh and abdomen; afterwards with partial palsy of the respiratory muscles, giving rise to a violent dyspnœa; and lastly, with amaurosis of the right eye and with loss of smell. The patient ultimately recovered from all her infirmities.

It has been often asked, how comes it that the extensor muscles appear to be always more affected by the paralytic weakness than the flexor—as we may fairly presume to be the case, seeing that paralytic limbs are almost always in a semi-flexed position. M. *Gabrini*, in speaking of the saturnine form of the disease, accounts thus for the phenomenon in question. Supposing, says he, that the enfeebling effect of the lead poison acts equally upon both sets of muscles, we should remember that the extensors require for their action a greater degree of force than the flexors, in consequence of the latter having their points of insertion into the bones at a less acute angle than the former; and being therefore more favourably placed for moving the joints. Such being the case, we are not surprised that the extensors are the first which indicate a diminution of contractile power; and, as the action of the flexors is not correspondingly diminished, the result is that the affected limb becomes more or less completely bent.

The following curious case occurred in the practice of M. *Piorry* some years ago. A young English girl was subject to hysterical attacks every month. As each attack subsided, she became affected with some form or another of paralytic weakness. At one time it was hemiplegia; at another time there was so complete an anæsthesia of certain limb or limbs that needles might be forced pretty deeply into the flesh without giving pain. Sometimes the sense of hearing was entirely gone; then she became blind, or the patient lost her speech; and thus it was that scarcely a single part of her body escaped being affected with a loss of power. Occasionally all these phenomena exhibited themselves together: there was an abolition, more or less complete, of the sensorial and locomotive faculties; the power of organic life and of the mind remaining quite intact. She usually recovered from these attacks in the course of a day or two. On one occasion she remained an entire month in this sad state; then, all on a sudden, she felt a sort of tremor in her head, as if a fluid ran along the spinal marrow, and all the morbid phenomena vanished, as if by enchantment.—*Annales Medico-psychologiques, Janvier* 1844.

### AGUE, A DISEASE OF THE NERVOUS SYSTEM.

The following remarks are taken from the same paper by Dr. *Macario* as those in the preceding article. The subject of hysterical Paralysis is therefore partially resumed.

"It is worthy of notice—although the fact has usually been much overlooked—that Hysterical phenomena are sometimes complicated with intercostal Neuralgia; and it is a singular circumstance that, when this Neuralgia is situated on the left side of the chest, the patient is often affected with symptoms of Ague; and that when it is on the right, agueish symptoms are wanting, and the patient suffers from what has been called hepatic Colic—which indeed is only one form of neuralgia.

"These phenomena, if examined in all their bearings, may throw some light on the ætiology of Intermittent Fevers. Have we not reason to believe that the element, so to speak, of these fevers is of a nervous nature? The very circumstance of their exhibiting paroxysms and intermissions—a feature that is strikingly characteristic of the class Neuroses—naturally leads us to this conjecture. If any organic lesion were present, (as some writers have most erroneously supposed,) think you that there would be every now and then so complete a cessation of all the febrile phenomena, that the patient and his friends might reasonably fancy for a time that the disease was entirely eradicated? We should think not. There might indeed, in the case supposed, be a recrudescence of the febrile symptoms every evening and morning; but then they would nevertheless continue with greater or less distinctness and severity during the intervals.

"Among the long catalogue of diseases, none, but those of the class Neuroses, are liable to those regularly-recurring exacerbations and remissions. And the occurrence of paralysis, which is sometimes observed to appear and vanish with the febrile paroxysm—*Torti* and others mention such cases—may fairly be adduced as an argument in support of this view. There are therefore strong reasons for believing that the morbific element in Intermittent Fevers is of a nervous nature."

(There is certainly much in the history of Agues to warrant this view of their ætiology. Besides the arguments adduced in the foregoing extract, we might point to the facts of Neuralgia being a not unfrequent sequela of such fevers; and of this neuralgia being often of an intermittent character itself, and being curable only by the use of anti-periodic medicines. Besides these considerations others might be quoted. For example, how much are both diseases under the influence of the mind and feelings! Often has it been known that a resolute effort of the will alone has served to prevent or arrest an expected paroxysm of Ague, as well as a threatened attack of Neuralgia: and every physician knows full well that a change of air and scene will generally suffice to work an almost instantaneous change for the better—provided, as a matter of course, the diseases have not existed so long as either to have induced any organic or humoral change, or to have materially deteriorated the general powers of the system.)

---

### OCCASIONAL TROUBLESOME CONSEQUENCES OF WEARING EAR-RINGS.

After the wound which is made in piercing the ears, there happens sometimes a particular kind of affection, which has not been, that we know of, noticed by surgical writers. It is a fibro-cartilaginous tumor, which has for its root the cicatrix of the wound made in the lobe, and which, in course of time, may acquire a considerable size. We have had occasion to observe three cases of this kind during the last few years; and each time the tumor was formed on the posterior side of the lobe. The first case occurred in a girl, ten years of age, whom I saw in the year 1839; the second, in a girl of eight years, and the third, in a young woman of four-and-twenty years of age, corpulent, and of a lymphatic constitution: for some weeks before she was married, she had been much distressed at the thought of appearing at her wedding without ear-rings; and she came, in the Spring of the year 1842, to beg me instantly to perform an operation that might enable her to do so.

The affection was well marked in all the cases, but particularly in the first girl, of whom we shall now speak briefly. There was on the posterior side of the lobe of the right ear a globular tumor, mammillated, and nearly of the form, and size of an ordinary hazel-nut; it was of extreme hardness, covered with a thin skin, adherent, of a natural colour, and on its surface were seen very minute venous ramifications; its base, attached to the lobe, adhered to it only by a root which traversed its entire substance; the tumor itself ending in a small cicatrix, which was on its anterior surface. On pressing the anterior surface of the lobe against the tumor, this root might be felt, and its extreme hardness was discovered; indeed, this prolongation was only the cicatrix of the wound made in the substance of the lobe when the ear was pierced.

In each of these girls, the presence of ear-rings had caused such pain as to oblige their parents to remove them very shortly after having had them put in.

In the second case, the tumor was of a less regular form, and its root was larger. Her parents formerly had had the tumor shaved clean off, and the ear pierced a-fresh to replace the ear-rings; but the child could not bear them, and the tumor re-appeared. The bride also had made several trials of this kind, but always without success; the root of the tumor gradually became larger, and acquired in course of time a prismatic form.

In each of these cases the removal of the tumor was effected by first cutting the tumor clean away on a level with the surface of the lobe, and then enucleating its root by two elliptic incisions. The edges were brought together with a stitch, and the wound healed up without accident. The cicatrix did not serve for the re-application of ear-rings, as the parties had desired, and they were advised not to make a new puncture till a much later period.

On examining these tumors, it was found that the texture of the body and the root was of a fibro-cartilaginous nature.

In the month of November last year, I saw, in the Edinburgh Infirmary, under the care of Professor *Syme*, a woman who had, upon the lobe of the right ear, an affection in some respects analogous to that of which we have been speaking, but which, nevertheless, essentially differed from it. In this case the entire thickness of the lobe was hypertrophied, and had undergone a fibro-cartilaginous degeneration. The lobe was amputated by making two flaps which, being held together by two sutures, healed quickly.—*Annales de Chirurgie.*

---

### Spontaneous Rupture of the Inferior Cava.

A young soldier, of a robust constitution, was seized on the 30th of December with pain in the abdomen, chiefly in the umbilical region; it was not increased on pressure, nor accompanied with any other marked symptom. The medical officer of the regiment regarded it as *enteralgic*, and prescribed rest and a gentle aperient. As the patient was no better in the evening, the cupping-glasses were applied on the right side of the abdomen, and gave immediate relief. He was left asleep at night, and early next morning was found dead in his bed. It deserves notice that this man had enjoyed excellent health up to the day when he was seized: the day before, he had mounted guard as usual.

*Dissection.*—The abdomen was much distended, and exhibited numerous dark patches of discolouration. On dividing its parietes, a large quantity of blood flowed out. It was long before we could find out whence it had proceeded. At length it was discovered that there was a rent—large enough to admit the point of the finger—in the inferior cava, on the level of the last dorsal vertebra: its edges were irregular and fringe-like. All the viscera were quite healthy.

It follows from the pathological appearances in this case discovered on dissection, 1st, that the death must have been rapid; and 2nd, that the perforation of the vena cava was probably consecutive upon an atrophy of the parietes of this blood-vessel.

The opinion of M. *Vidal (de Cassis)* on this subject is thus expressed in his "Traité de Pathologie Chirurgicale et de Medecine Operatoire":—"Atrophy, when extending to each of the membranes which compose a blood-vessel, or even affecting but one of them, necessarily weakens it, and sometimes reduces the vascular parietes to an extreme tenuity. Hence arises the fear of these ruptures, which have been observed in the superior cava, the vena portæ, &c. These ruptures are either with or without some appreciable morbid lesion."

M. *Andral,* in his "Precis d'Anatomie Pathologique," cites a case in which, in the middle of a scuffle, a man in good health fell down suddenly, and expired in a few seconds. On opening the body, there was found a perforation of the abdominal vena cava. The edges of this perforation seemed as if they had been torn; but around it, the vessel appeared in a perfectly healthy state.—*Annales de la Chirurgie.*

### TREATMENT OF BED-SORES.

A writer, in a recent number of *Walther* and *Ammon's* Journal, recommends the application of a lotion composed of equal parts of spirits of Camphor and the vegeto-mineral water of Goulard. The parts, that have become red by the pressure, should be repeatedly moistened with this lotion; it requires to be briskly shaken before it is applied.

If, in spite of this treatment, the skin should break, the zinc or lead ointment, to which some camphor has been added, is a good application. In still more obstinate cases, an ointment, consisting of four parts of fresh-prepared Tannate of Lead, and thirty of lard, has been sometimes found to answer extremely well. On the whole, however, nothing succeeds so uniformly, alike as a prophylactic remedy against the abrasion of the skin and a healing one to that which has become broken, as a solution of Creosote—prepared after the method of *Reichenbach*—in the proportion of one part of the oil to 80 parts of water.

When the affected part becomes gangrenous, fomentations with a decoction of Yellow Bark, to which some tincture of Myrrh has been added, may be useful. Some patients have found benefit from the sprinkling of the ulcerated surface with a powder composed of Bark, Camphor, and Myrrh; others, from the use of the Camphorated styrax ointment. The tinct. Benzoini compos., or Friar's Balsam, is often an excellent application to bed-sores. Whatever be the nature of the application employed, the most important remedy of all is the removal of pressure from the affected parts, by means of air or water cushions. The comfort derived from the use of these is most pleasing.

### EXCISION OF THE ENTIRE SCAPULA WITH A PORTION OF THE CLAVICLE.

An old soldier, who had served in the Imperial Guard during the campaigns of

1813, 1814, and 1815, had been for several years distressed with uneasiness and pain in the upper part of his left arm. In the course of time, a hard tumor formed there; being attached, it was thought, to the humerus: it was extirpated; and the wound healed kindly. Six months afterwards it was deemed necessary to amputate the limb at the shoulder-joint, in consequence of the cervix of the bone having become decidedly diseased. The round head of the humerus, as well as the surface of the glenoid cavity, was at this time most attentively examined and pronounced to be entirely free from any morbid alteration. This operation also succeeded perfectly. Little more however than half a year after its performance, the patient began to experience pains in the cicatrix, and ere long a new tumor formed in the site of the axilla. As this increased in size, the pains became more severe: it had already attained the bulk of the closed fist, when the case again came under the observation of Professor *Rigaud* of Strasbourg. The swelling was evidently of an osseous nature, and seemed to be attached to the cervix of the scapula; it was free from any adherence to the parietes of the chest, following all the movements of the shoulder: the clavicle seemed to be quite intact. After a tedious dissection the entire scapula, having the tumor affixed to it, was extirpated, the clavicle having been first cut across with a chain-saw passed under it. The cure was complete in the course of two months.—*Comptes rendus de l'Academie.*

---

### Hæmorrhage after Lithotomy, checked by Creosote.

During the performance of this operation on a boy 14 years of age, it was remarked that, when the skin and perineal muscles were divided, there was a more profuse discharge of blood from the wound than usual. After the calculus had been extracted, the hæmorrhage nearly ceased for a couple of hours; but then it returned as profusely as ever. From its character, M. *Daser* suspected that the blood proceeded rather from a multitude of minute vessels than from any single one of considerable size. Several small arteries had been tied during the operation; and cold applications and absolute repose were now enjoined. These failing, the wound was plugged up with a piece of sponge; but, after a few hours interval, the blood having accumulated within the bladder, violent expulsive efforts came on and forced the sponge and a large quantity of coagula out. A canula was then introduced by the wound and retained in the bladder, and the sponge was replaced; still the bleeding continued. As the patient had now become very pale and faint, M. *Daser* had recourse to the local use of creosote. He dipped a sponge in the pure oil, and inserted it deep into the wound, having previously secured a piece of tape to it, so that it could be easily withdrawn at any time. For a few minutes the pain induced by the application was very sharp, but then it subsided altogether. The edges of the wound assumed a blackish-grey colour, and the hæmorrhage ceased. At the end of ten minutes, expulsive efforts ensued, and the sponge, along with a quantity of coagula, was expelled. A fresh sponge was introduced; and no further discharge of blood took place. M. *Daser* was for some time afraid lest the application of the creosote might induce a gangrenous inflammation of the deep-seated parts; but fortunately no such accident followed. Next day the sponge was again expelled, but without any renewal of the bleeding. Every thing went on well, and the wound was entirely healed by the end of the third week.

The styptic action of the creosote was well marked in the present case. Although highly irritating and almost caustic, the secondary inflammation that followed was very trifling.

M. *Daser* suggests its application to atonic, varicose, and other unhealthy ulcers.—*Graefe's and Walther's Journal.*

### Discussion at the Royal Academy respecting Ophthalmia.

A few months ago, there was a very lengthened and most elaborate discussion on this subject among the learned members of the French Academy. It was continued during at least five or six sittings, and the Journals of the day exhibited to the reader's eye page after page of the most wearisome, and often too most contradictory, assertions. As is generally the case with field-day harangues, but little light seems to have been thrown upon the question at issue by the numerous and long-spun speeches that were delivered. All, that we propose to do, is to give a brief summary of the speeches of the members who opened the discussion.

M. *Velpeau* read a report upon a memoir by Dr. *Morand* of Tours, on an epidemic of Scrofulous Ophthalmia, which had prevailed among the sick prisoners of the Colony at Mettray. The disease had resisted every mode of treatment, until Dr. M., observing that it always commenced with an inflammation of the pituitary membrane, fortunately thought of applying the nitrate of silver to this part, by introducing up the nostrils an ointment—composed of from one to two parts of the salt to 20 parts of lard and almond oil. This plan was followed by the most pleasing results.

M. *Velpeau* expressed his regret that Dr. M. had not employed an anatomical denomination in place of the very faulty one of *Scrofulous* Ophthalmia. Since he had read this memoir, he had examined the state of the pituitary membrane in a great number of cases of Ophthalmia; but he had not found any traces of inflammation on it, except in a very few cases. In ordinary circumstances therefore, the treatment of ophthalmia by any application to the nostrils he considered to be certainly not admissible: the practice may however, he thought, be useful in certain cases.

M. *Roux* desired to learn from the reporter for what reason, and to what extent, he objected to the appellation of Scrofulous Ophthalmia. Does he mean to say that we should not designate ocular inflammations according to the nature of their causes? and that we should have respect only to their seat, and the tissue or tissues which are most affected? Does he wish us to believe that any one mode of treatment,—that by the application of caustic for example—is equally applicable to each and all of them, whether the disease have a syphilitic, a scrofulous, a rheumatic, or any other origin?

M. *Castel* remarked that a *Scrofulous* Ophthalmia cannot be said, properly speaking, ever to be epidemic; for there is no such thing as an epidemic form or variety of scrofula. It is quite a misnomer to attribute the character of *scrofulous* to an epidemic ophthalmia.

M. *Velpeau* said that he had no wish or intention to suppress the appellative qualifications of scrofulous, syphilitic, &c. superadded to the terms which designate the inflammations of different parts of the eye. All that I mean to affirm, he continued, is that we cannot properly, in the present state of ophthalmological science, call one of these inflammations merely scrofulous, and nothing more; this appellation is not sufficient; we must indicate with greater exactitude the tissue that is affected, whether this be, for example, the conjunctiva, the cornea, &c., and then we may add an epithet to characterise its special nature. We should therefore speak of scrofulous conjunctivitis, scrofulous keratitis, &c., instead of employing a vague general term, as that of scrofulous ophthalmia for all inflammations of the eye.

M. *Gerdy* spoke to the following effect. "The German spirit, which is dominant in the present day in ophthalmic medicine, is essentially retrograde and antiprogressive. Our neighbours divide and subdivide diseases in the most arbitrary way, alike without reason and without measure. The eye is an organ of exceedingly small dimensions, not exceeding 12 or 13 lines in diameter in any direction. Surely therefore it is difficult to conceive how inflammations, whose character on all occasions is to spread, can be confined to any one of the tissues of an organ, which is altogether so small. The innumerable divisions, endeavoured to be established in the voluminous works of the German writers, do not exist in Nature. I have no hesitation in saying that it is utterly impossible to distinguish such a host of inflammations, such as the spirit of system has attempted to describe. I do not mean to assert that the inflammatory action may not be confined to some one of the parts or tissues of the eye, when it is not severe; moreover I am ready to admit that it may commence in one part or tissue rather than in another; but what I maintain is, that the entire organ necessarily becomes in course of time more or less implicated in the morbid action, as soon as this acquires any degree of intensity. Read, if you have the patience to do it, the fastidious symptomatology that you find in the works of pathological writers, and you will meet, for each variety of ophthalmic inflammation, with a series of phenomena that it is utterly impossible to distinguish at the bedside of your patient. Then again as to the treatment to be pursued, this is generally the same in all cases. But it is not sufficient to point out faults and defects; we must endeavour to show how they are to be avoided and rectified. In my opinion, all that requires to be done is to describe the inflammation of the whole eye, indicating in the most simple manner how this morbid state may be confined in some cases to a single point, in other cases to two points, and so forth. As a matter of course, due attention must be paid to the idiosyncrasy and peculiar constitution of each patient."

M. *Velpeau* admitted that the infinitude of distinctions enumerated by German oculists is most unnecessary; but he did not go so far in his objections to their style of writing as the preceding speaker. I do not think, said he, that we should reject them all, as he seems to do. Although the anatomical elements are accumulated in the eye, it does not follow that they may not be affected with inflammation separately, at least in the earlier stages of the disease. Any one, who has attentively studied the diseases of the eye, can have no difficulty in distinguishing conjunctivitis, keratitis, iritis, &c. It is quite true that each of these inflammations requires the same remedies for their cure; but this holds true of all inflammations. Shall we therefore say that their particular diagnosis is unnecessary, and of no utility? Certainly not; for, though the general treatment may be alike in all, the local remedies that are required in each are different.—*Comptes rendus.*

---

### Hypertrophy of the Cartilages of the Trachea and Bronchi.

Professor *Gintrac*, of Bourdeaux, relates the following case in a recent number of the Montpelier Journal of Medicine.

A boy, eight years old, and of a delicate ailing constitution, had been from his infancy affected with a greater or less degree of dyspnœa, which was occasionally accompanied with sharp pains in the chest, and other symptoms of thoracic distress. He had been repeatedly admitted into the hospital; and had generally received relief, at least for the time, from bleeding and the application of blisters between the shoulders. When he entered the hospital on the 24th of May last, his breathing was exceedingly oppressed, so as sometimes—more especially in the evening—to threaten suffocation: there was however no cough present. Auscultation discovered nothing, except a strong sibilant noise in the bronchi; and every part of the chest was perfectly resonant on percussion. There was nothing abnormal in the pulsations and sounds of the heart, save that the former were somewhat more obscure than usual; the pulse too at the wrist was regular. The abdomen was rather full, but the evacuations were healthy.

This state, of which the mos prominent symptom was a constant dyspnœa—

every now and then liable to great aggravation—had been but little relieved by the remedies that had been employed, such as enemata of Assafœtida, fumigations of Stramonium, the use of Calomel, Sulphuret of Soda, &c. The child left the hospital on the 8th of July, but was brought back 17 days afterwards; the symptoms then being unusually intense. The suffocative dyspnœa continued up till the period of death, which occurred on the 8th of August.

*Dissection.*—The body was much emaciated, the sternum was very prominent, the thorax was fuller on the left than on the right side, and the face and extremities were œdematous. The lungs were found to adhere to the thoracic parietes, except at the upper part on the left side, where there was present a small quantity of limpid serosity: in every other part the pleura, whose tissue was much thickened throughout, was more easily detached from the ribs and the intercostal spaces than from the surface of the lungs. The larynx was healthy, and so seemed the trachea likewise externally; but, on slitting it open, its parietes were discovered to be much thicker and harder than usual. This alteration was most conspicuous at the lower part, close to the point of its bifurcation: it extended also to the bronchi, for a considerable extent. The result was, that the calibre of the air-passages was very sensibly smaller and more contracted than in a state of health. The lungs were œdematous; for, on dividing them, a quantity of yellowish serosity oozed from the divided surfaces: the lower lobe on the right side was hepatised.

---

### Periodic Swelling of the Knee cured by Opium.

A middle-aged woman, of intemperate habits, sprained her left foot in February, 1843. Six weeks after the accident, and when it seemed that she had quite recovered from its effects, the ankle-joint and lower part of the leg became suddenly very much swollen, hot and painful: this state continued for a few hours, and then gradually subsided. The swelling, &c. on the next and following days recurred about the same time of the day—two o'clock; and, after lasting for about three or four hours, disappeared. The general health remained perfectly good during all this time.

The periodicity of this affection, and the absence of all inflammatory symptoms, induced me, says the reporter, to order quinine, which was administered to the amount of 15 grains daily: the joint was, at the same time, ordered to be rubbed with a camphorated opiate liniment. These means were steadily continued for six days; but no benefit was produced. The starched bandage was then tried, with the view of preventing the recurrent swelling; but the use of it caused so much pain, that it was at once discontinued. Trial was next made of the affusion of cold water upon the joint, followed by brisk friction: the internal use of sudorifics and colchicum was conjoined with this local treatment; but everything seemed to be utterly impotent. On finding this, I began the use of the Acetate of Morphia. Its effects on the complaint were very speedily manifest: each day the pain and swelling became lets considerable, so that, in the course of a week, the patient appeared to be perfectly well.

On the day however following that when she had discontinued the use of the Morphia, the swelling and other symptoms again made their appearance at about ten o'clock in the forenoon, and continued for nearly the usual time. I wished now to give another trial to the quinine; it was therefore administered in large doses, but again it failed. The Morphia was then taken, and with equally decided effects as on the former occasion. Its use was continued for a fortnight; after which it was suspended, without any return of the symptoms.—*La Belgique Medicale.*

*Remark.*—We saw a case, the other day, in which the right ankle was every now and then the seat of swelling and severe pain extending up the leg and disabling the patient—who was a young delicate girl—from walking. From the character of the symptoms, constitutional as well as local, we regarded the affection as of an hysterical nature, and therefore prescribed steel and bitters. The relief thus obtained was speedy and decided.—*Rev.*

### Magnetisation of a Steel Rod by the mere Influence of the Will!

M. *Thilorier*, on the 11th and 18th of June last, endeavoured to establish, by a series of experiments before the French Academy of Sciences, the existence in the human body of a fluid or agency analogous to the electric, and capable of producing some very curious phenomena. Many facts, he said, might be adduced to prove this position, which will probably startle most who have not thought of the subject. Of these, one of the most satisfactory is that of magnetising a steel rod, placed upon the epigastrium, by the mere and unaided effort of the will! By the simple act of volition, this effect is produced; for it is well known that the mere contact of the rod with the surface of the body is not sufficient. M. *Thilorier* says that there are three principal points of the body from which the magnetic fluid escapes, viz. the hands, the epigastrium, and the forehead! In a state of repose or passiveness of the intellect and will, as for example during sleep, the effluvium, and consequently the magnetisation, are not however entirely null. In the hands, the currents of the fluid are parallel to the direction of the fingers and perpendicular to the palm; those of the epigastric and frontal regions are directed from below upwards, and from the feet to the head, in a curved line! The magnetic action is null, or nearly so, at the lower end of the spinal marrow; it increases in degree or intensity in proportion as we approach the neck! Currents emanate from every point of the cranial vault; but the most energetic of all is from the culminating point of the frontal bone! The energy of the currents depends on a variety of circumstances: it is more considerable about noon than at night, or in the morning; likewise more so in health than in sickness!—*L'Experience.*

### Treatment of Hydrocele with Ioduretted Injections.

In more than 300 cases of this complaint treated with an ioduretted injection, (composed of tincture of iodine 4 parts, and distilled water 125 parts,) by M. *Velpeau*, not a single accident or unpleasant symptom has ever occurred. One of the patients indeed died; but the fatal result in this instance proceeded from a purulent inflammation of the cellular tissue of the pelvis, quite unconnected with the operation, and not having any communication whatever with the affection of the scrotum. The average period for effecting the cure was 15 days. In one case only the injection found its way into the tissue of the scrotum, in place of the tunica vaginalis: notwithstanding this misadventure, no appearance of gangrene supervened, and the patient recovered without any unpleasant accident.—*Ibid.*

### Treatment of Fistula Lachrymalis.

The practice that has been followed, for many years past in Italy, in the treatment of chronic inflammation of the Lachrymal Canal is to introduce a minute fragment of the *lunar caustic* into the fistula, if such be present; or by a small puncture made into the sac, if not. An acute inflammatory action is immediately set up; but by the following day this is usually much diminished, and ceases entirely in three or four days afterwards. The engorged parts then gradually subside, the purplish colour changes to a white, the tears resume their natural course, and every trace of the disease vanishes. In most cases a single application of the Caustic is sufficient. Professor *Lallemand*, we are told, very generally adopts this mode of treatment.—*Clinique de Montpelier.*

---

### Passage of Metallic Mercury into the Blood and various Organs of the Body.

M. *Oesterlen* has performed a number of experiments on animals, with the view of determining this point. The results of these are as follow:—

1. It is indubitable that mercury may pass, in the metallic state, through the parietes of the blood-vessels, since minute globules of it have been found in the subcutaneous cellular tissue and in the veins permeating it. The globules have never been discovered in the epidermic layers, but only in the deep-seated layers of the dermis, near the blind extremities of the hair-follicles; also in these follicles and in the sudoriferous canals. 2. The metallic mercury, rubbed on the skin or introduced into the intestinal canal, may give rise to injurious effects, by passing into the current of the circulation. It is not easy to determine in what manner the metallic mercury, when once introduced into the circulation, becomes changed and modified, or how it then acts. At the side of the shining globules, M. *Oesterlen* always found a number of dull and dark-coloured corpuscles, which resembled a good deal the granules of a mercurial oxyde; these were found to be not acted upon by alkalis, but to be dissolved slowly in strong nitric acid, after being ground down into a fine powder. In the urine and in the bile, the mercurial globules did not exhibit any appearance of decided change. 3. Minute globules of this metal, in the state of fine division, may traverse the capillaries without producing any inflammatory *stasis:* their presence in the vessels does not seem to influence the formation of the blood or the development of the sanguineous corpuscles. 4. Small quantities of mercury, taken inwardly or applied on the skin, appear to pass chiefly into the parenchymatous substance of the spleen, liver and kidneys, and to be discharged by the last two emunctories.

M. *Oesterlen* has never been able to detect the presence of any globules in the *cells* of the liver, or in the corpuscles of the spleen, or in the minute tubercles of the kidney: they were always on the outer surface of these organs. He conjectures that they may have escaped from the anatomical preparation, after it had been put up. He has, however, detected them in the saliva and also in the urine. —*Roser's Archives.*

---

### On the Quantity of Carbonic Acid exhaled from the Lungs in Twenty-four Hours.

The results of M. *Scharling's* experiments on this subject are the following:—

1. The quantity of carbonic acid exhaled from the lungs varies considerably in different periods of the day.

2. These variations are attributable, in part, to the varying power or faculty of the respiratory organs to change the inspired air into the gaseous product; and in part also to the unequal movement of the circulation—the state of which is very sensibly affected by that of the process of digestion.

3. *Cæteris paribus*, a person exhales a greater quantity of carbonic acid when full than when he is fasting, and also more during waking than sleeping.

4. Men exhale more than women of the same age, and children more than adults.

5. In various states of sickness, there is considerably less exhaled than during health.—*Schmidt's Jahrbucher.*

---

### On the Epidemic Intermittance of Intermittent Fevers.

The development of Agues in marshy countries is by no means uniform or constant: it is itself subject to intermittances. Thus, at Antwerp and its environs, in 1822 and 1823, these fevers began to become more common and severe than they had been observed to be for some years before. Their intensity increased during the following seasons.

"The periodical genius or type," says M. *Gouzee*, "arrived at its acme in 1826, the period of the memorable epidemic of Grouingen. During the three summer months of that year, which were remarkable for an almost constant dry heat of from 20° to 28° Reaumur, the number of insidious and malignant remittent fevers was considerable at Antwerp, among all classes of the population. During the month of July, twenty-five, thirty, and even forty fever cases entered the military hospital daily. In 1827, this epidemic constitution, although still very decided, was nevertheless not of so great violence; after having suffered a little remission in the following years, it re-appeared, and prevailed again with considerable intensity in 1834, 1835, and 1836.

"During this long succession of years, more particularly in the first eight or ten, nothing was so common as marsh cachexias, leucophlegmatic inflammations and engorgements of the spleen. It was not uncommon to meet with invalids in whom the hypertrophied spleen occupied the entire left side down to the pubis. The frequency of malignant fevers at that time obliged the medical men to be constantly on the watch. In 1837, a rapid change took place all at once: the intermittent fevers ceased, and their sudden disappearance coincided with the appearance of a severe epidemic of Influenza, which prevailed from the middle of January to the end of the following month. During the entire prevalence of this new epidemic, we did not meet with a single case of intermittent fever—a circumstance well worthy of notice.

"From 1837 to 1841, that is to say during an interval of five years, the paroxysmal fevers were so rare, and so slight, that the sulphate of Quinine, formerly the anchor of safety in the majority of cases, had then in a manner fallen into neglect. The malignant remittent fevers, the obstructions of the spleen and the marsh cachexias had also almost entirely disappeared. At last, the periodic fevers re-appeared in 1842; and, during the following year, in our localities, they returned to such an extent and often with such gravity as could not fail to arrest the attention of all our practitioners. During the months of August and September of this year, the appearance of a good many cases of pernicious fevers was noted at Antwerp; a circumstance which, for more than six years before, had not been met with in practice."

These variations proceed, according to our author, from dry prolonged heats, without great agitations of the air, and cold nights.

"In our low and marshy countries," he observes, "it is not, as many physicians believe, the humidity of the atmosphere that occasions the development of intermittent fevers. There is no situation in which fewer paroxysmal fevers are met with when the seasons, in which they generally shew themselves, are rainy and damp. If the humidity of the air is necessary to their development, it is in districts not so low as ours, in order to prepare the work of miasmatic, decomposition, which it requires other conditions of the atmosphere to complete."—*Journal Belge.*

*Remark.*—From the tone of the preceding observations, our readers will perceive that medical men on the Continent are beginning to pay attention to a subject, connected with the history of diseases, which has been far too much neglected in the present century—we mean the nosological influences of seasons, atmospheric changes, and so forth. We need not say that the writings of *Hippocrates, Sydenham, Baglivi,* &c. are pregnant with allusions to this matter.

### THE BEST MEANS OF PREVENTING THE MARKS OF SMALL-POX.

*Zimmerman* and *Rosen* were the first to make it generally known that the development of variolous pustules might be much arrested by the application to the skin of mercurial plasters. This fact had been nearly forgotten, when M. *Serre,* and subsequently MM. *Briquet* and *Nonat,* drew the attention of the profession to the value of this mode of treatment, to which the appellation of *ectrotic* has been applied. According to their experience, nothing answered so well for this purpose as the *emplastrum Vigo*—a mercurial preparation somewhat similar to the *emplastrum hydrargyri cum ammoniaco* of the London Pharmacopœia. The recent experiments made by *Coppen* confirm and give additional value to the remarks of these gentlemen. He found that, if common mercurial ointment be applied to the papules of small-pox from their first and earliest appearance, they do not suppurate, but speedily contract and dry off: this ointment however has not the effect of preventing the eruption of the papules, however early in the disease it may be applied to the skin. But the *emplastrum Vigo* he has found to have superior efficacy; for, according to his observations, it serves to arrest the eruption of the papules, as well as to prevent the suppuration of those that have already appeared.

Dr. C. has also tried the effects of an ointment containing sulphur, as an application to the skin in Variola; but the results of his experience are certainly not favourable to its use.—*Annales de la Soc. Med. de Gand.*

---

### TREATMENT OF THE ITCH IN BELGIUM.

The following Circular has been addressed to military surgeons by the Inspector-General of the Belgian army.

"Each patient is to be supplied with an ounce or an ounce and a half of liquid sulphuret of lime in a small pot: this quantity he is to rub carefully and slowly with his hands on every part that is covered with papulæ. If there be any papulæ on the back, another patient is to rub the liquid upon that part. The operation is to be repeated three times in the twenty-four hours, so that each patient consumes three or four ounces of the sulphuret daily. A bath is to be taken every alternate day; the frictions are to be suspended on that day. Fifteen frictions (or ten days use) are usually sufficient for the cure of the disease, if the medical officer in charge sees that the remedy is properly used."

The sulphuret is prepared thus: take of sublimed sulphur 16 pounds, and of quick lime 32 pounds: boil in 80lbs. of water for three-quarters of an hour. Let the mixture rest for some time until it settle, and then let the clear fluid be decanted off. Boil the residue afresh in about the same quantity of water, treat it in a similar manner, and add this decoction to the first. Usually 140 pounds of the sulphuret, at 12° by the areometer, are thus obtained. If the liquid be more dense, it should be lowered to this standard by the addition of rain water. —*Ann. de la Soc. de Med. d'Anvers.*

### Cases of Poisoning with Cantharides.

The practical question as to the best mode of treatment in this accident is well illustrated by comparing the following two cases with each other.

*Case* 1.—A lunatic, 45 years of age, swallowed about half an ounce of a paste which contained nearly two drachms of powdered cantharides: this was about half-past seven o'clock in the morning. In the course of a quarter of an hour afterwards, attempts were made to provoke vomiting by administering tartar-emetic, and ipecacuan in hot water. Copious vomiting was speedily induced, and the patient rejected so much of the ingested mass, along with much of the mucus of the stomach, that it was believed that very little of the poisonous matter was left behind in the stomach. Towards the after part of the day, the patient's body was found to be very cold, and his pulse at the same time was contracted and rather indistinct. A slight re-action ensued, and this state lasted for about twelve hours, when symptoms of adynamic depression returned, and next day the patient died about eight o'clock. The only treatment, that had been adopted, was the free use of mucilaginous and other emollient drinks. This case is related in the *Giornale delle Scienze Mediche di Torino*, and is thus commented upon by the French reviewer in the l'Experience.

"It is highly probable that the fatal termination in this instance might have been prevented, if, in place of trusting to the use of such inefficient means as soothing diluents, the medical men had pursued what is commonly called the *medicine of symptoms*—the only rational method, it may be observed, to combat the constitutional phenomena of poisoning. Two circumstances in the preceding case rendered it favourable for successful treatment: 1. The one was the copious rejection of the contents of the stomach by vomiting; and 2, the little soluble form in which the poison existed. The paste had not remained sufficiently long for it to become dissolved in the juices of the stomach and absorbed into the system. The fatal result may therefore be attributed in part to the adoption of an erroneous treatment. The physician in the next case understood his duty better, and the consequence was that he saved his patient."

*Case* 2.—A healthy and middle-aged man swallowed a scruple of powdered cantharides, which had been mixed with his food by some friends in sport. In the evening he felt a sense of burning and constriction in his mouth and throat, also nausea and frequent efforts to vomit, shiverings and tremor in his limbs, &c. The general weakness and depression became greater and greater; the twitches, pains and formication in the limbs and back were more troublesome; the urine was passed with great difficulty and scalding; there was a distressing tenesmus; the thirst was intolerable, and the extremities were continually icy cold. During this (the second) day nothing but acidulated and demulcent drinks was administered; but, as the symptoms became gradually more alarming—for paralysis of the lower extremities, and bloody micturition had now come on,—Dr. *Podrecca* was called in. At this time the patient's countenance was pale and livid, his eyes were hollow and dark, and his pulse was exceedingly low: he complained of intense anxiety at the præcordia, occasional priapism, severe pains in the abdomen, &c. Wine, spirit of canella and laudanum were immediately ordered to be given in frequently repeated doses; so that, in the course of twelve hours or so, the patient took 5 *litres* of the first, 4 ounces of the second, and 4 scruples of the last. The unfavourable symptoms gradually decreased, and next day the patient voided his urine pretty freely—which he had not been able to do for thirty-six hours. The weakness and cramps in the lower limbs continued for some time afterwards; but these also subsided in the course of a few days.—*Annali Univ. di Medicina.*

*Remarks.*—If our readers will turn back to the Number of this Journal for April 1839, they will find some very interesting observations, by Dr. *Giocomini* of Padua, on the subject of poisoning with Cantharides. He shews that this substance exerts a powerfully contra-stimulant and hyposthenic effect on the system; and therefore that the proper remedies to be administered, in cases of poisoning with it, are stimulants and cordials.

Besides this article, there is another in one of the subsequent numbers, wherein M. *Orfila* (we believe) has described a good many experiments where animals were poisoned with Cantharides; and in these also it was found that the chances of recovery were always in proportion to the freedom with which stimulants had been administered.

The fear of administering stimulants in cases of poisoning with acrid substances is too common. It should be remembered that the great object of treatment, in such circumstances, is to combat and overcome the *tendency to death*; and, therefore, that our practice should be regulated by the nature of the symptoms that are present, and not by our suspicions of the pathological lesions that may have been induced.

---

### A Tænia evacuated through an Opening in the Abdominal Parietes.

*Bremser, Rudolphi, Cruveilhier* and others, have questioned the accuracy of the recorded histories of lumbrici making their way through the parietes of the intestines; but the more recent researches of M. *Mondiere* would seem to prove indisputably the perfect authenticity of the alleged fact, and to shew—

1. That Lumbrici may work themselves a passage through the parietes of the intestines, by simply separating the fibres of these parietes by means of their head or anterior extremity;

2. That, by reason of the contractility of the muscular fibres of the intestinal parietes, the opening, which has given passage to the worm, is immediately obliterated, and leaves no perceptible trace behind;

3. That, in consequence of this arrangement, a verminous tumor, when it opens outwardly, cannot communicate directly with the cavity of the intestines;

4. That a verminous tumor may be formed at any point of the abdomen, but that it is most commonly developed about the inguinal and umbilical regions.

Instances of the discharge of Tæniæ in this manner are of much more rare occurrence. Only two cases are on record, as far as we are able to ascertain. One is related by *Mouleng* in the Journal de Medecine, tome LVI., and the other by *Sporing*, in the Memoires de l'Acad. des Sciences de Suede, tome IX. In both these cases, however, the presence of fæcal matters in the verminous tumor, and the permanence of a fistula after the discharge of the tænia through the abdominal parietes, induce the belief that the worm had made its way outwardly through an *ulceration* of the bowels.

The following fact, recorded as it is with all necessary details by Professor *Siebold*, serves to make it probable that a *tænia* may traverse the parietes of the intestines by merely separating their fibres, in the manner that *lumbrici* are in the habit of doing.

A young man was received into the surgical hospital in consequence of several scrofulous abscesses on different parts of his body. A tumor, which was supposed to be of this nature, occupied the umbilical region. A few days after his admission, Dr. *Herz* was in all haste summoned to the patient, as the swelling had suddenly burst, and given issue to a tænia, which was alive and moved about quite freely. It was cautiously laid hold of, and slowly and very gradually drawn out to the extent of several ells. No gas, chyle, bile or fæcal matter escaped from the opening; in short, there was nothing to lead to the suspicion that the verminous swelling had any communication with the cavity of the intestines.—*Medicinische Zeitung fur Heilk. in Preussen,* 1843. No. 17.

---

### M. Cazenave on Alopecia, or Baldness.

The causes of this lesion or defect are numerous and varied. It is very common after small-pox and other severe febrile affections; also in the course of phthisis and other protracted diseases. Whatever attenuates and exhausts the powers of the system, is sufficient to induce it. Thus, it is not unfrequent among prisoners and the inmates of workhouses, gaols, &c.; and who does not know that intense or long-continued mental application and moral emotions of a depressing character cause the hair to fall off, as well as change its colour from dark to sober grey?

Baldness appears to be sometimes owing to a syphilitic taint. There has been a good deal of difference of opinion among medical men on this point; but M. *Cazenave* thinks that there cannot be a reasonable doubt on the subject. And let it be understood that he does not allude merely to the form which has been attributed to the action of mercury on certain constitutions, nor yet to that which not unfrequently occurs when there is an actual venereal eruption, whether tuberculous or squamous, present on the scalp at the time; in such cases there is not any reason to attribute the falling off of the hair to the constitutional *nature* of the disease, rather than to the local presence of the tubercles or pustules. But what M. *Cazenave* means by Syphilitic Alopecia is a genuine secondary symptom, which may occur without any co-existing farinaceous exfoliation, or other manifest disease of the cutaneous tissue. This species of Alopecia is not so rare as is generally imagined. Among other cases, M. *Cazenave* mentions one that may be worth noticing. It occurred in a middle-aged man, who had been affected with gonorrhœal inflammation of the testicle. At the end of four months, and without the appearance of any other secondary syphilitic symptom, the patient lost almost all his hair, which was strong and of a dark colour. The Orchitis remained for a long time imperfectly cured; a relapse of the disease occurring every now and then. When recourse was had to the steady use of mercurial frictions, it was observed that the hair of the head ceased to fall off.

Alopecia is not unfrequently caused by the cutaneous affections which have been called *herpes tonsurant, porrigo decalvans* and *pityriasis capitis,* and by other squamous diseases. The scalp becomes the seat of an inflammatory action, which betrays itself sometimes by a more or less copious formation of scales, and at other times by the eruption of red papulæ or patches, which are followed by the exfoliation of the cuticle. This dermoid inflammation does not affect either the bulb or the piliferous tube; but the hair, being surrounded and girt, at its passage through the skin, by inflamed tissues, so as to be compressed in a sort of squamous channel, becomes dry and friable, and then breaks off; in this manner the part becomes gradually bald either spontaneously, or in consequence of over-combing and brushing it. Such is the nature of *pityriasis capitis* —an affection, by the bye, that is perhaps the most frequent in those women whose hair is dark, long and thick. It is very generally aggravated by the use of those very means, that are used to remove the scurf and scales. M. *Cazenave*

has often had occasion to observe it in ladies who suffer much from neuralgic headaches. As the pilous bulbs remain unaffected, the hair often grows again; but only, at least in most instances, to fall off with greater readiness than before.

All the acute diseases of the hairy scalp—from Erythema and Erysipelas to Eczema and Impetigo—may induce a more or less complete state of baldness; and, as the dermoid inflammation sometimes penetrates to the piliferous tubes, the capsules or bulbs may eventually become seriously affected. This is especially apt to be the case in those impetiginous eruptions that are known by the name of *tinea*. As a general remark however it may be asserted that the baldness, which supervenes upon any acute affection of the scalp, is of short continuance, and that it rarely and only occasionally happens that the bulbs are destroyed, or the piliferous tubes obliterated. To this form of the disease, we may refer the baldness that is often induced by venereal eruptions—a variety of the disease that is very different, as we have already noticed, from the genuine syphilitic Alopecia.

The baldness, that ensues upon *tinea favosa*, seems to be owing to the obstruction of the piliferous tubes, and to the consequent obstacle that is presented to the passage of the hairs through them. Hence it often happens that, during the continuance of this disease, the scalp over a considerable extent exhibits a white and shining aspect, without any distinct follicular orifices, and with perhaps only a few scattered hairs. Now this state of the skin cannot at all properly be regarded as a cicatrix of the skin, but rather as a new tissue, very thin in substance, devoid of colour, and having, in consequence of the obliteration of the sebaceous orifices, lost its natural polish. But, if the piliferous tubes have been obliterated, the bulbs have not been destroyed. Hence we can easily discover with the lens, if not with the naked eye, the hair which, under this sort of transparent cicatrix, is about to issue at several points from the external envelope which it cannot pierce, and under which it is sometimes seen to bend upon itself, until it becomes atrophied and the bulb along with it.

The causes of Baldness being so various, the treatment of the complaint must necessarily be different in different cases. The first thing to be done is to determine whether the skin be actually diseased or not. If the falling off of the hair co-exists with a healthy state of the integument, the cause of the complaint must be sought for in the general or constitutional condition of the patient. Its nature is very often manifested by the existence of certain concomitant symptoms, such as the presence of headaches, certain exanthematous eruptions, &c. If there be any local affection of the scalp, the diagnosis, at least for all practical purposes, is obvious: it is to the existing eruption that our attention must be directed; for the baldness is only the result of it; and, by removing the cause, the effects will gradually pass away.

When we have reason to believe that the bulbs of the hair are fairly atrophied or destroyed, it is evident that we cannot reasonably expect to remove the baldness. If this be the effect of a deficient secretion, and the expression of a particular constitutional condition, the case will require a two-fold plan of treatment, one local and the other general. Repeated shaving of the head should be enjoined; for this process seems not only to produce a very beneficial local excitement, but also to render the function of secretion more active.

In the baldness, which sometimes supervenes upon tedious recovery from accouchement, as well as upon fevers and many chronic maladies, we have only to use one or two very simple remedies, as for example dry rubbing of the scalp, to induce the re-growth of the hair—never forgetting, as a matter of course, the restoration of the general strength by appropriate treatment.

Very different is that which is required in that form of Alopecia which is accompanied with the loss of the colour in the scalp—as we see in cases of *vitiligo* and *porrigo decalvans.* Here we must trust almost exclusively to special remedies; such as stimulant ointments and lotions—the immoderate and ill-timed use of which, in other forms of the complaint, so often does much more harm than good.

In Syphilitic Alopecia, the internal use of mercury or of the Ioduret of Potassium is necessary for the cure.

Whenever there is an exanthematous eruption present on the scalp, the remedies applied must be suited to the nature of the existing local affection, according as this is inflammatory or not. In *Pityriasis Capitis*, the local affection is usually of an inflammatory nature, and it must be treated accordingly. A very common and pernicious error is, that in such a case we should endeavour to excite the energy of the bulbs, and stimulate the secretion of the hair by those very means, which, by aggravating the inflammatory action, necessarily increase more and more the evil they were expected to relieve. All irritating applications should be avoided; and, when the inflammatory condition of the scalp is corrected, we may then with advantage have recourse to the use of an ointment composed of a little borax and lard, blended well together: a weak alkaline solution also will be found useful in many cases. All sorts of warm and tight head-dress should be most religiously avoided; and benefit will generally be obtained from the regular use of tepid bathing.—*Annales des Maladies de la Peau.*

---

### M. Lisfranc on Hæmorrhoidal Tumors.

Hæmorrhoids cannot properly be regarded as *erectile* tumors. They are formed by a sort of fibrous tissue which is traversed by blood-vessels, that are liable to become every now and then more than usually swollen and congested. However great the congestion may be, the vessels in hæmorrhoidal swellings are never so numerous as in properly-so-called erectile tumors.

In several thousand cases, says M. *Lisfranc*, in which I have extirpated the lower part of the rectum, I have scarcely ever met with a truly erectile tumor in this part.

The non-erectile nature of hæmorrhoidal tumors renders our prognosis much less unfavourable, and renders unnecessary, in the treatment of them, the operation that is usually indispensable for the cure of erectile tumors.

As to the *treatment* of hæmorrhoids, we have to attend to many circumstances for the regulation of our conduct. If they are in a state of inflammation or painful congestion, we should first determine whether the symptoms be remittent or permanent. In the former case, we should be on our guard not to check suddenly any discharge of blood from the anus, more especially in such habits as are of an apoplectic tendency; for, indeed, a hæmorrhoidal hæmorrhage often seems to serve the same purpose in the male system as the catamenial does in the female. If, however, the pain becomes very severe, or if the hæmorrhage is so profuse as to demand the interference of the medical man, the question occurs: should we, as is so generally done, have recourse to the application of leeches to the anus? This treatment will certainly answer tolerably well on some occasions; but most frequently the local congestion is thereby aggravated rather than remedied. What we should do in such circumstances is to draw blood from the arm to the extent of ten, twelve, or sixteen ounces, on the first day; and perhaps repeat the operation to the extent of four or six ounces on the following. The use of a warm bath will much promote the good effects of the bleeding. If the introduction of the canula up the rectum does not produce much pain, an enema of nearly cold water, to which a few drops of laudanum should be added, is a very useful remedy. No poultice should ever be applied to the anus, as it only serves to increase the congestion of the already over-swollen vessels.

When the hæmorrhoidal tumors are protruded through the anus and are constricted and perhaps partially strangulated by the sphincter, how should the surgeon proceed to reduce them? It is generally recommended that he first smear his fingers with oil or some other unctuous substance. Such an advice must surely have been given by one, who is not acquainted with practice;—for the protruded tumor is usually too slippery, and we require to wipe its surface before attempting to replace it. The object and endeavour should be to push gently backwards and upwards, first, that portion of the gut which was last protruded, and so on gradually until the whole is re-introduced. If the patient strains much during this operation, his attention should be distracted as much as possible.

Let us now briefly notice the treatment of hæmorrhoidal tumors when they are in a chronic condition. If they are readily reducible and are not attended with pain or any great inconvenience, they are generally excised, without much consideration as to the risk which the patient may thereby run; for it never should be forgotten that the extirpation of hæmorrhoids is not wholly devoid of danger. For myself, I generally avoid operating in such circumstances; and, being convinced that surgery, when it effects a cure without the loss of blood, is a thousand times more brilliant, I avoid, as frequently as possible, having recourse to the use of cutting instruments. For the purpose of diminishing the size and fulness of hæmorrhoidal tumors, I enjoin a mild and soft diet, cooling drinks, and occasional spoliative or derivative bleedings. When this treatment is insufficient, I find an excellent adjuvant in directing a stream of cold water upon the anus, or even up the rectum. We often effect a cure by this means; and in almost every case the general health of the patients becomes, at the same time, decidely improved. When we cannot command the use of the *douches*, we have found a useful substitute for them in gently touching the surface of the protruded hæmorrhoids with the nitrate of silver, with the view of stimulating and not of cauterising them. The *bourrelets* usually shrink up in consequence of the excitation induced, and do not again protrude.

If the hæmorrhoidal tumors exhibit simple ulcerations, they should be cauterised with the nitrate of silver, or with the acid nitrate of mercury. If the ulcerations are of a decidely unhealthy nature, and the tissues be indurated, the diseased part should be excised. To guard against the excessive, and sometimes fatal, hæmorrhage that may occur after this operation, I recommend the following plan:—let the hæmorrhoidal *bourrelet* be first laid hold of and drawn out by a tenaculum, and surrounded by two elliptical incisions; in place then of removing it by one or two sweeps of the knife or scissors, it should be detached slowly by successive strokes at different points of its circumference. By adopting this method, the blood-vessels, as they are divided, are prevented from being retracted upwards: they can therefore be secured either by ligature or by torsion. When I come to the point of the circumference of the *bourrelet* at which I begun, I lay hold of the mass to be divided between my thumb and fore-finger, in order to find out if it contains any artery which may be easily commanded; and then I detach it very cautiously and leisurely, dividing it by successive strokes of the bistoury. Even this operation is, it must be confessed, tedious, painful, and occasionally followed by fatal consequences.—*Gazette des Hôpitaux.*

